# VNIR Hyperspectral Signatures and Machine Learning for Early Detection and Classification of Barley Diseases

**DOI:** 10.3390/plants15121854

**Published:** 2026-06-15

**Authors:** Rimma M. Ualiyeva, Mariya M. Kaverina, Anastasiya V. Osipova

**Affiliations:** Department of Biology and Ecology, Toraighyrov University, Pavlodar 140008, Kazakhstan; ualiyeva.r@gmail.com (R.M.U.); aanastasiyaaa@internet.ru (A.V.O.)

**Keywords:** hyperspectral imaging, proximal hyperspectral sensing, UAV-based remote sensing, barley, spectral signatures, machine learning, automated disease classification, phytosanitary monitoring

## Abstract

This study focuses on identifying barley diseases at various stages using the unique spectral signatures of phytopathogen infections. We examined the causal agents of widespread crop diseases, including: loose smut, head blight, fusarium head blight (FHB), stem rust, net blotch, spot blotch, common root rot. Analysing disease-specific spectral characteristics with machine learning (ML) algorithms revealed the most informative spectral ranges: the green region (~520–560 nm), the red chlorophyll absorption zone (~650–680 nm), and the red-edge region (~700 nm). These ranges accurately reflect alterations in the plant’s cellular structure and pigment complexes. Spectral data were processed using five ML algorithms. Random Forest (RF) proved to be the most effective for identifying and differentiating barley diseases, achieving an accuracy of up to 90.13% (MCC = 0.86). This superior performance stems from the ensemble method’s robustness to noise and its ability to extract critical features from high-dimensional hyperspectral data, particularly when distinguishing diseases with overlapping spectral signatures. Furthermore, this study highlights the potential of integrating UAV-based remote sensing to delineate reference zones, proximal hyperspectral imaging (HSI), and ML for robust plant health monitoring. This combined approach shows significant promise for early disease diagnostics, enabling site-specific treatments, curbing disease progression, and reducing pesticide application. Ultimately, these findings offer practical value for the agro-industrial sector in major grain-producing countries, especially in Central Asia, where agricultural advancement is a strategic priority for sustainable development and food security.

## 1. Introduction

Barley (*Hordeum vulgare* L.) is one of the most important cereal crops in global agriculture. Its yield stability and grain quality depend heavily on effective phytosanitary monitoring, balanced nutrition, and the early detection of stress factors, including diseases. Agricultural intensification, climate change, and tightening environmental and quality standards demand high-precision, rapid, and automated diagnostics. Traditional methods remain labour-intensive, time-consuming, and often fail to detect diseases at early stages.

Remote sensing methods have emerged as a superior alternative, offering greater speed, objectivity, and automation. Recent studies demonstrate that hyperspectral imaging (HSI) quality—specifically spectral resolution and signal-to-noise characteristics—plays a critical role in the robustness of machine learning models applied to agricultural monitoring, particularly for disease classification in cereals [1]. HSI has proven highly effective for identifying seed-borne phytopathogens, including *Fusarium* spp., smut fungi, rust-related contaminants [2], yellow rust [3,4], leaf rust [5], and pathogen detection (*Fusarium*) using principal component analysis [6]. Furthermore, HSI enables the determination of moisture content [7], protein, starch, lipids [8,9,10], chemical composition in leaves and grains (N, P, K, Mg, Fe, Zn, etc.) [11,12], including nitrogen content [13,14], deoxynivalenol (DON) mycotoxin levels [15], melanin content [16], barley seed hardness [17], and nutrient concentrations in barley leaves [12]. The technology is also instrumental in discriminating between healthy and infected barley to quantify infection severity [18], assessing the toxic effects of benzo[a]pyrene on leaves [19], determining phenological growth stages [20], measuring phenolic compound content [21], estimating chlorophyll levels [22], and identifying barley varieties [23]. Combined with UAVs, HSI supports winter barley yield prediction [24]. Spectral signatures correlate with chemical changes (moisture, pH, organic acids, protein) [25], and red-edge and NIR reflectance relate to nitrogen content [26]. HSI is also valuable for early biotic and abiotic stress detection [27] in barley grains and leaves [28].

Multispectral imaging complements these capabilities through vegetation indices for yield prediction [29], crop differentiation (wheat vs. barley) over large agricultural areas based on spectro-phenological indicators, heading date and plant developmental stage determination [30], and chlorophyll estimation [31]. Machine learning and neural networks further enable precise crop segmentation and classification. Classification approaches employ Support Vector Machines (SVM), Random Forest, neural networks [32,33], Partial Least Squares Regression (PLSR) [23,34], Partial Least Squares Discriminant Analysis (PLS-DA) [35], Convolutional Neural Networks (CNNs) [36], and both pixel- and object-based methods [37]. Optimised CNNs achieve >94% accuracy [15] for barley disease classification (>90%) [38] and seed sorting by variety, quality [39], or protein content [40]. Models trained on wheat transfer to barley with up to 80–88% accuracy [36], while SVM and PLS-DA exceed 99% accuracy in detecting gluten contamination of oats caused by wheat, barley, and rye grains [41]. PLS-DA and PCA-LDA models identify non-viable barley, wheat, and sorghum grains at >90% accuracy [42]. Integrating HSI with AI markedly improves early pathogen detection, including fusarium and rusts [43], and reliably distinguishes sprouted from unsprouted grains [44].

Despite these advances, studies specifically focused on spectral characterisation and early disease detection in barley remain scarce. Applying HSI and machine learning to address this research gap holds immense practical value, offering pathways to reduce yield losses, optimise pesticide application, and strengthen agroecosystem resilience. This study aims to characterise spectral signatures of barley diseases at early developmental stages, identify the most informative wavelength ranges, and develop robust classification models to enhance phytopathological diagnostic accuracy and reliability.

## 2. Results

### 2.1. Selection of Informative Hyperspectral Data Components and Spectral Signatures of Barley Diseases

**Figure 1:**
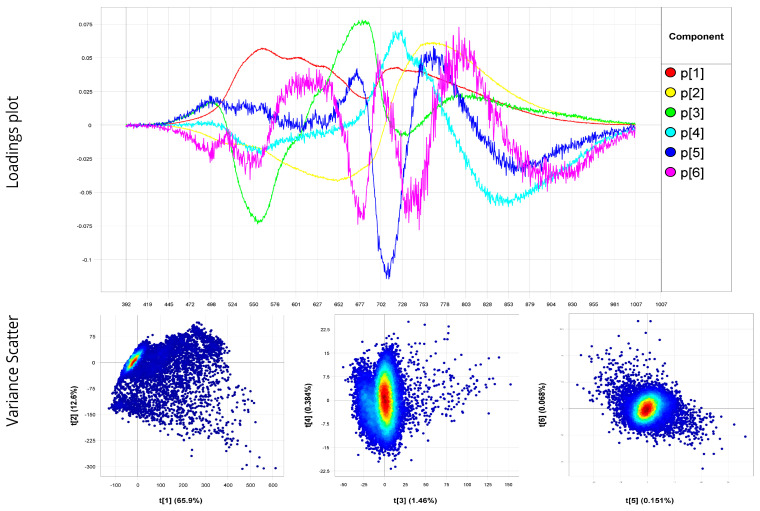
Principal component loading coefficients and variance plots for loose smut.

**Figure 2:**
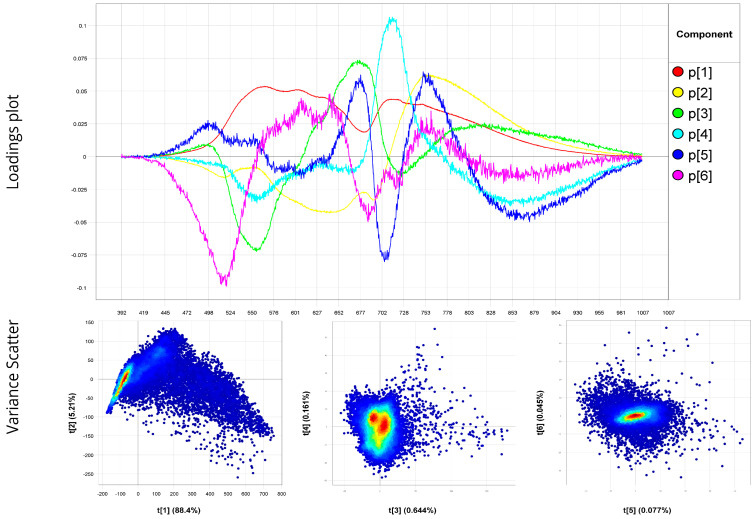
Principal component loading coefficients and variance plots for head blight.

**Figure 3:**
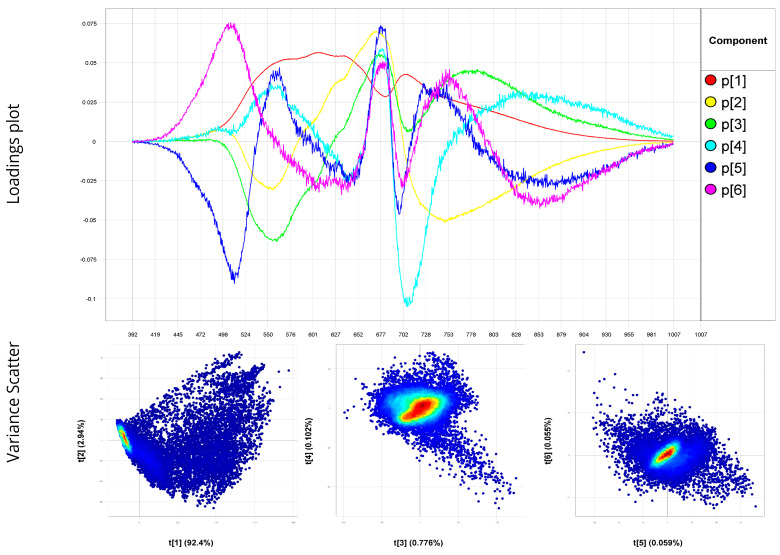
Principal component loading coefficients and variance plots for FHB.

**Figure 4:**
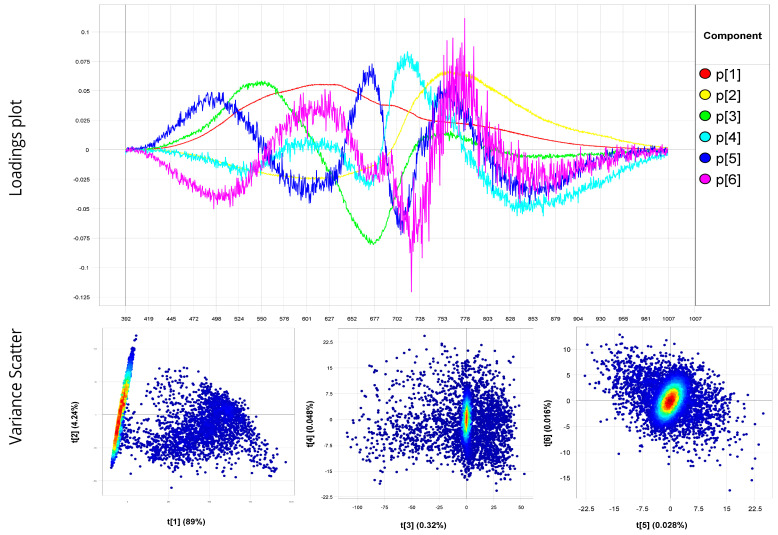
Principal component loading coefficients and variance plots for stem rust.

**Figure 5:**
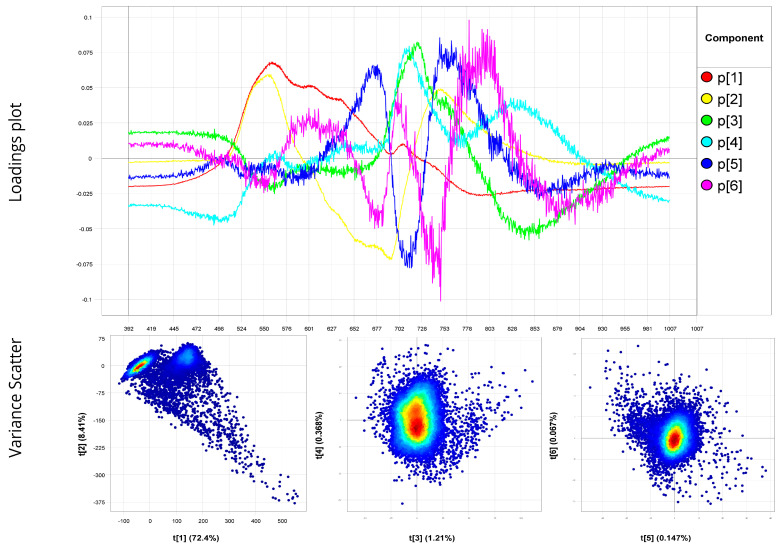
Principal component loading coefficients and variance plots for net blotch.

**Figure 6:**
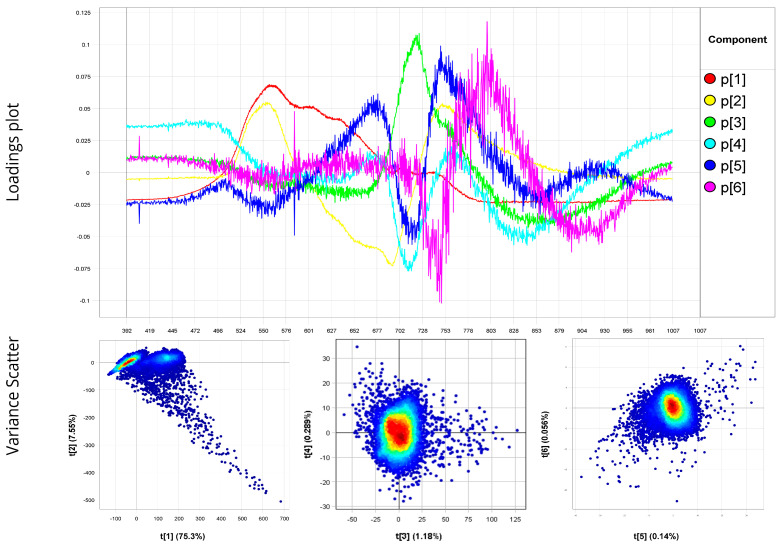
Principal component loading coefficients and variance plots for spot blotch.

**Figure 7:**
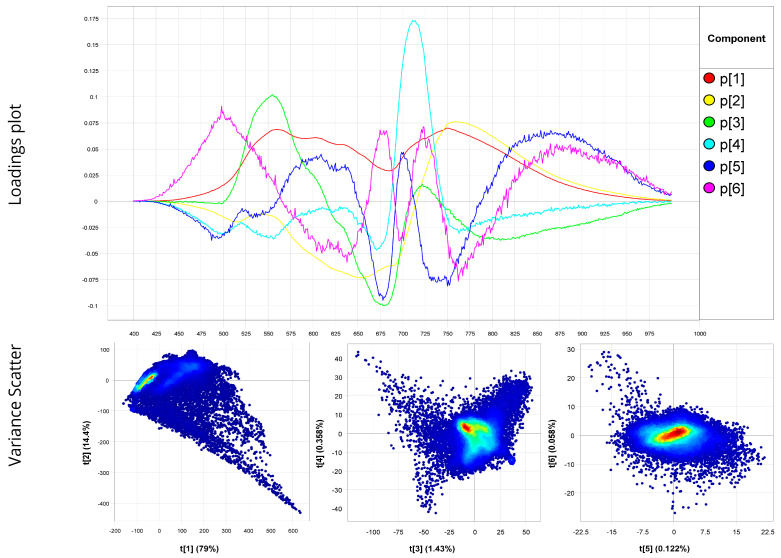
Principal component loading coefficients and variance plots for common root rot.

The most informative principal components of the hyperspectral images were selected based on each component’s contribution (loading) to the total data variance. Figure 1, Figure 2, Figure 3, Figure 4, Figure 5, Figure 6 and Figure 7 present the loadings across the working wavelengths and the pixel distribution of each component in principal component space.

Loading plots display the loading coefficients for principal components t[1]–t[6] as a function of wavelength (400–1000 nm), where each curve represents the contribution of a specific wavelength to the corresponding principal component. Variance scatter plots show sample distribution in principal component space (Figure 1, Figure 2, Figure 3, Figure 4, Figure 5, Figure 6 and Figure 7).

Analysing the variance scatter diagrams for components one through six (Table 1) revealed that PC1 and PC2 were the most significant (Table 2). Notably that the values of explained variance and cumulative variance reported in Table 2 represent mean values calculated across all studied barley disease classes. Since PCA was applied consistently to each disease-specific dataset under identical preprocessing conditions, Table 2 provides an aggregated representation of the overall spectral variance structure, whereas Table 1 presents disease-specific variance distributions. This approach ensures comparability of principal component contributions across different phytopathological conditions.

**Figure 8:**
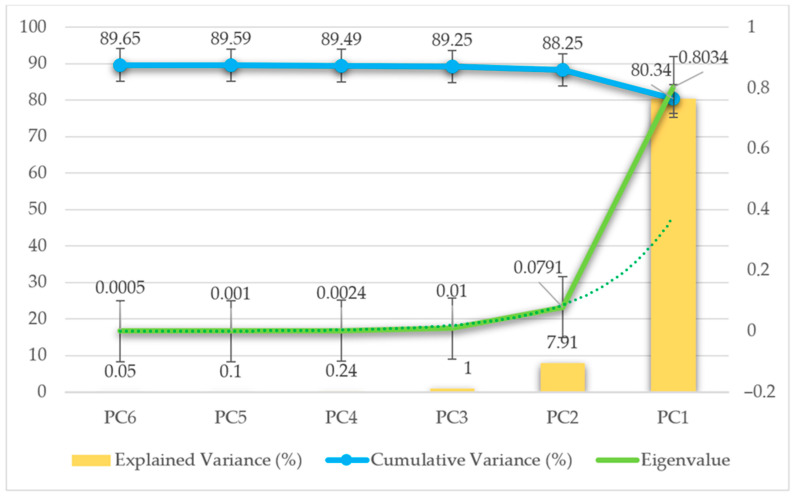
Scree plot of principal components showing explained variance, cumulative variance, and eigenvalues.

PC1 and PC2 were identified as the most informative components (cumulative variance = 88.25%), justifying their selection as the primary focus for hyperspectral image analysis. Their high informativeness is confirmed by the explained variance values (PC1 = 80.34%, PC2 = 7.91%) and eigenvalues (PC1 = 0.8034, PC2 = 0.0791) (Table 2, Figure 8), as well as by the loading coefficient analysis for each component (Figure 1, Figure 2, Figure 3, Figure 4, Figure 5, Figure 6 and Figure 7, Table 3).

**Figure 9:**
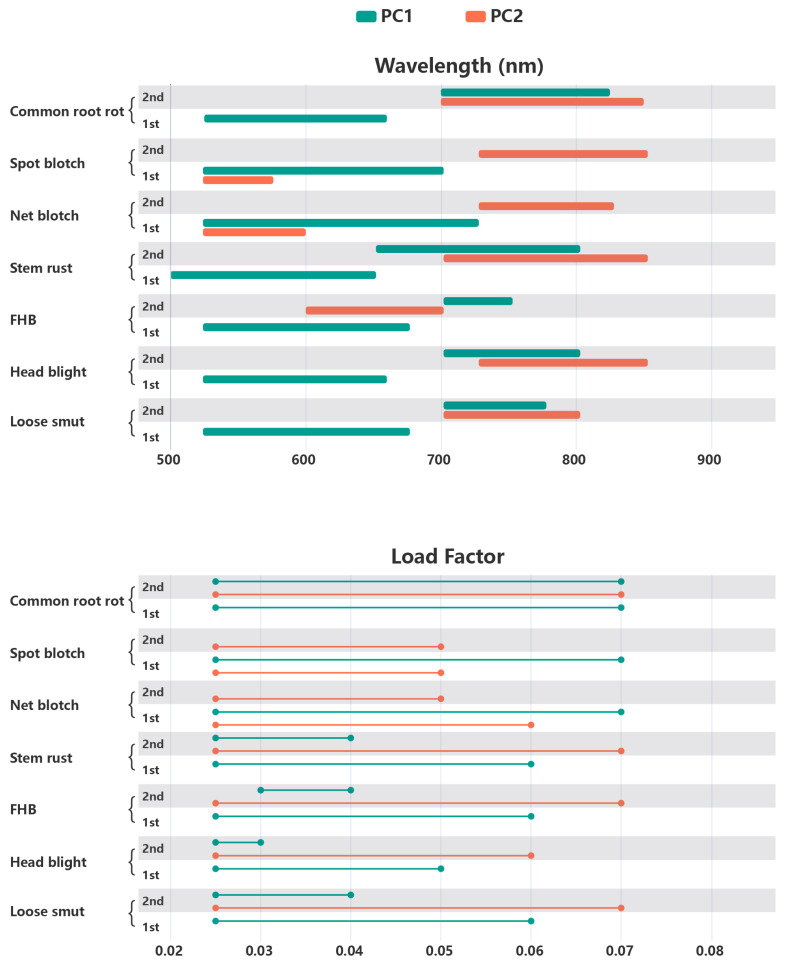
PC1 and PC2 factor loadings in the VNIR range for spectral features of barley diseases.

Table 3 and Figure 9 present the factor loading ranges and their distribution across wavelength regions for spectral features of barley diseases. A clear division into two stable spectral zones was observed for all diseases: the visible range (500–700 nm), which dominates the first peak of both components, and the red-edge to near-infrared range (700–850 nm, in some cases 652–853 nm), mainly associated with the second peak. Across all diseases, the first peak consistently occurred within 500–677 nm, while the second peak was typically located in the 700–803 nm range. The only exception was the second peak for FHB, which appeared at 600–702 nm. Overall, these results indicate a stable partitioning of informative spectral regions and confirm the relevance of the selected components. Factor loadings for all diseases ranged from 0.025 to 0.07, suggesting high comparability of feature contributions. This indicates that differences between barley diseases are driven primarily by the spectral location of lesions rather than by differences in loading magnitude, as loadings serve as normalised indicators of feature importance. The presence or absence of specific peaks (the second peak in PC1 for net blotch and spot blotch, and the first peak in PC2 for loose smut, head blight, FHB, stem rust, and common root rot) reflects the distinct pathogenesis of these barley diseases.

**Figure 10:**
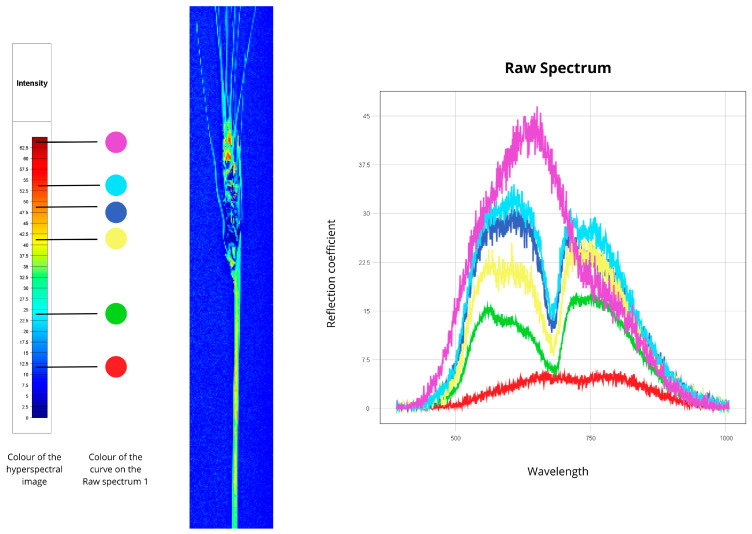
Spectral profile of a barley plant infected with loose smut (*Ustilago nuda*).

**Figure 11:**
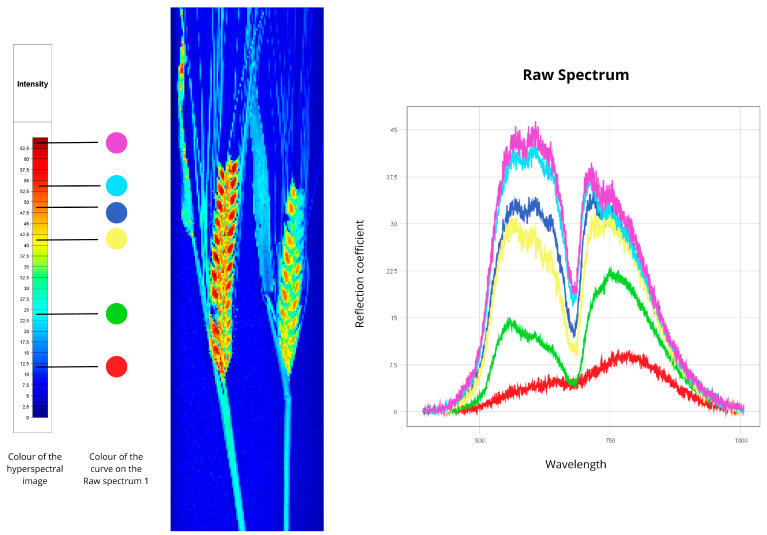
Spectral profile of a barley plant infected with head blight (*Bipolaris sorokiniana*).

**Figure 12:**
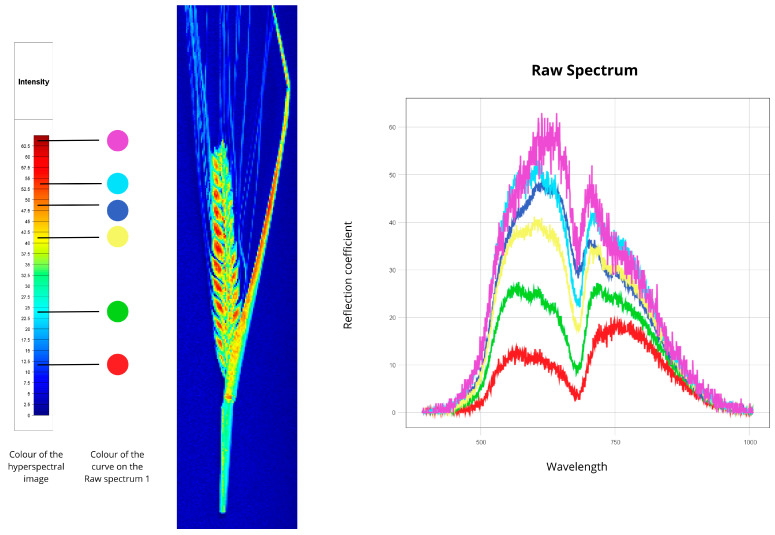
Spectral profile of a barley plant infected with FHB (*Fusarium* spp.).

**Figure 13:**
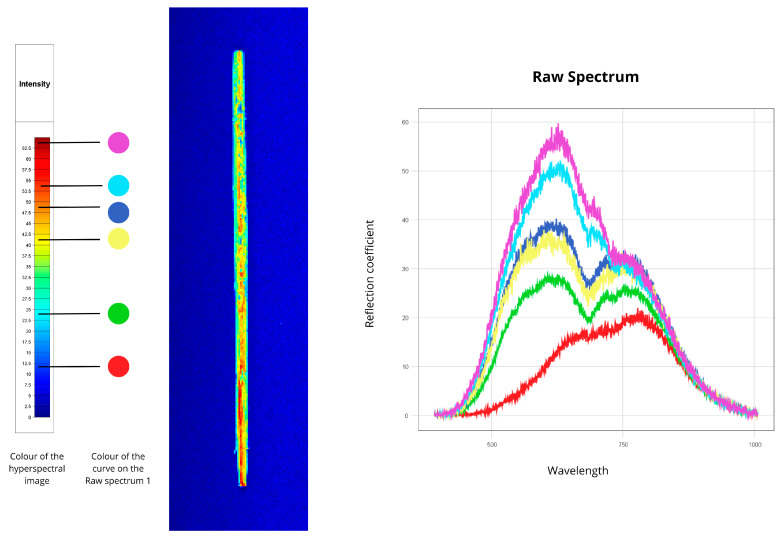
Spectral profile of a barley plant infected with stem rust (*Puccinia graminis*).

**Figure 14:**
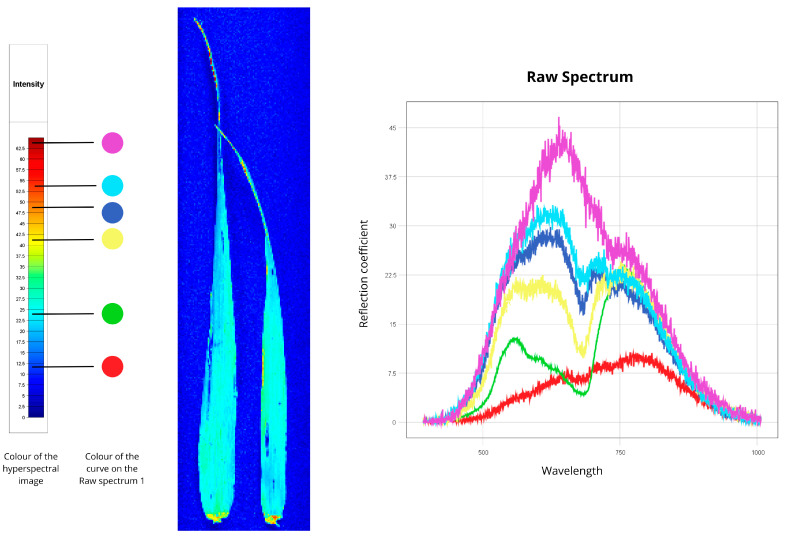
Spectral profile of a barley plant infected with net blotch (*Pyrenophora teres*).

**Figure 15:**
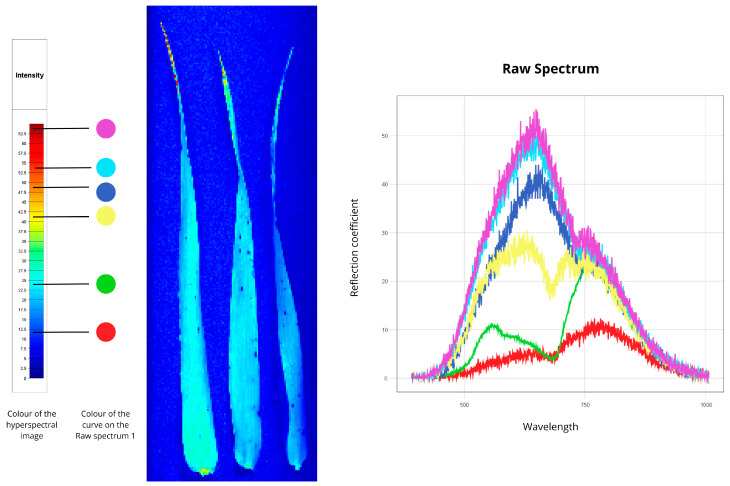
Spectral profile of a barley plant infected with spot blotch (*Bipolaris sorokiniana*).

**Figure 16:**
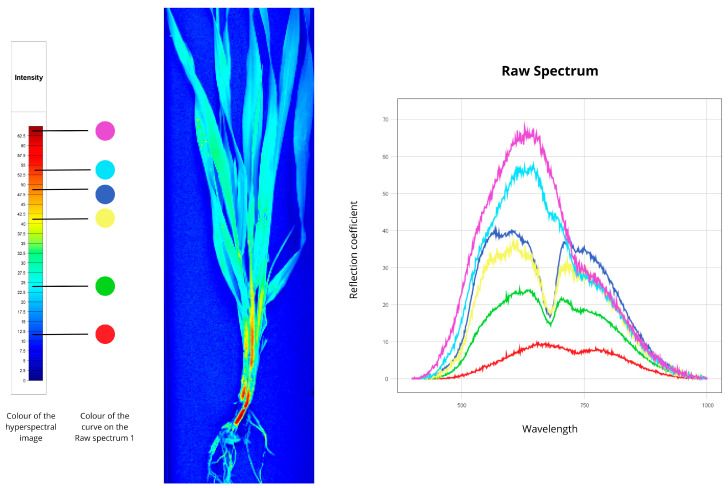
Spectral profile of a barley plant infected with common root rot (*Bipolaris sorokiniana*).

Figure 10, Figure 11, Figure 12, Figure 13, Figure 14, Figure 15 and Figure 16 present hyperspectral images of barley infected with various disease pathogens and their corresponding spectral fingerprints.

#### 2.1.1. Loose Smut (*Ustilago nuda*)

Figure 10 shows a sample infected with loose smut. The red curve represents diseased areas, which appear as blue zones with reduced intensity in the hyperspectral image.

This curve exhibits no distinct peaks or red-edge response, maintaining a low reflectance of 7%. The purple curve corresponds to disease-induced desiccated spike areas, with a first peak at 45% reflectance in the 500–750 nm range and a weakly expressed second peak at 22.5% reflectance in the 750–780 nm region. The green, yellow, blue, and cyan curves relate to healthy spike tissues. Their first peak lies within 500–700 nm and the second within 700–780 nm. The green curve represents dehydrated awns (first peak: 15%, second: 17%). The yellow curve corresponds to the spike tip (first peak: 22.5%, second: 25%). The blue and cyan curves show first-peak reflectance of 30% and second-peak reflectance of 25%.

Figure 1 presents the loading plot and variance scatter for the loose smut-infected sample. The first principal component, p[1], is the smoothest curve with no sharp fluctuations, describing the main spectral variability (65.9% of variance). The second, third, and fourth components show moderate fluctuations and capture local spectral features associated with disease expression; their combined variance contribution is 14.44%. The absence of a pronounced red-edge response and the uniformly low reflectance values indicate extensive destruction of photosynthetically active tissues caused by fungal sporulation. These spectral characteristics suggest severe impairment of chlorophyll-containing structures and serve as reliable indicators for detecting advanced loose smut infections.

#### 2.1.2. Head Blight (*Bipolaris sorokiniana*)

Figure 11 presents a sample infected with head blight. The red curve represents diseased spike areas (shown in blue on the hyperspectral image). This spectrum is characterised by low overall reflectance across the entire range (7.5%), with no distinct peak in the visible region. The remaining curves exhibit two pronounced maxima: the first peak in the 500–700 nm range and the second in the 700–780 nm region, with a distinct red edge near 700 nm. The green curve corresponds to desiccated awns and leaf tissues (first peak: 15%, second: 22.5%). The yellow curve represents healthy spike areas with 30% reflectance at both peaks. The blue and cyan curves also depict healthy spike tissues, with first-peak reflectance reaching 35% and 38%, and second-peak values of 37% and 37.5%, respectively. The purple spectrum represents the most physiologically active, well-illuminated healthy spike areas, with a first peak reaching 45% and a lower second peak in the NIR at 37.5%.

In the loading plot (Figure 2), the first principal component p[1] shows a smooth, relatively broad curve with maxima in the visible 550–650 nm and 702–778 nm ranges, gradually decreasing in the NIR. The variance share of t[1] is 88.4%. The second and third components (p[2], p[3]) display more pronounced extrema with troughs around 700–728 nm. Head blight causes chlorophyll degradation and necrotic spotting, redistributing reflectance in the red region (~670 nm) and altering red-edge steepness (700–740 nm). These components capture changes in the pigment complex and tissue density, together accounting for 5.854% of variance. The variance scatter shows points elongated along the t[1] axis, confirming the dominant role of the integral spectral factor linked to overall infection severity. A dense core with an elongated tail indicates sample heterogeneity in pathological intensity. The observed reduction in reflectance and modification of the red-edge region are consistent with chlorophyll degradation and necrotic tissue formation caused by pathogen development.

#### 2.1.3. Fusarium Head Blight (FHB) (*Fusarium* spp.)

Figure 12 shows a sample infected with FHB. All curves display two distinct peaks: the first in the 550–700 nm range and the second within 700–780 nm. The purple and blue curves represent diseased spike regions and corresponding desiccated leaf areas, which appear red-orange in the hyperspectral image. For the purple curve, the reflectance coefficient is approximately 60% at the first peak and drops by roughly one quarter to 45% at the second peak. Similarly, the blue curve exhibits a first peak of 50% and a second peak of 40%. The cyan spectrum describes the transitional areas surrounding the fusarium lesions, with reflectance coefficients of 48% (first peak) and 35% (second peak). The remaining curves correspond to healthy tissues: the yellow spectrum shows reflectance values of 40% at the first peak and 35% at the second peak. The green curve exhibits a reflectance of 25% at both peaks, while the red spectrum corresponds to spike awns, where reflectance ranges from 10% at the first peak to 20% at the second peak.

In the loading plot (Figure 3), the first principal component p[1] has the smoothest shape with positive values in the 550–776 nm visible range and a gradual decline toward the NIR. This component describes overall reflectance and spectral changes driven by the pathogen’s cumulative impact, explaining 92.4% of variance—indicating its dominant role in the data structure. The second and third components (p[2], p[3]) show pronounced peaks in the 601–702 nm range. Alternating positive and negative peaks reflect differences between chlorophyll absorption regions (red band), photosynthetically active tissues (green region), and structural scattering effects in the red-edge region. In FHB-infected plants, these patterns are associated with chlorophyll degradation and tissue disorganisation caused by fungal invasion, which leads to redistribution of spectral reflectance across the visible and near-infrared ranges.

#### 2.1.4. Stem Rust (*Puccinia graminis*)

Figure 13 shows a stem infected with rust. The red curve represents isolated rust foci with reflectance values between 15 and 20%. The green curve describes tissues surrounding disease foci (first peak: 28%, second: 25%). The purple curve corresponds to desiccated leaf areas, with a reflectance coefficient of 58% within the first peak and 30% within the second peak. The remaining spectra represent healthy tissue. Specifically, at the first peak, reflectance reaches 35% for the yellow curve and 40% for the blue curve; at the second peak, these values drop to 30% and 33%, respectively. The first peak is typically located in the 500–700 nm range, while the second peak lies within the 700–780 nm range.

In the loading plot (Figure 4), the first principal component p[1] has a smooth, positive shape, particularly in the 524–778 nm range, gradually declining toward the NIR. The second and third components (p[2], p[3]) exhibit pronounced extrema in the 524–576 nm and 702–803 nm ranges. The spectral response of stem rust reflects the formation of uredinia and the accumulation of rust spores, which alter surface optical properties and reduce chlorophyll-related absorption.

#### 2.1.5. Net Blotch (*Pyrenophora teres*)

Figure 14 shows a sample infected with net blotch. The red curve represents diseased areas—appearing as blue zones with reduced intensity in the hyperspectral image—and exhibits no distinct peaks or red-edge response, maintaining a low reflectance of 7.5–8%. The purple curve reflects desiccated leaf areas caused by disease, with a first peak at 45% reflectance in the 500–750 nm range; the second peak is nearly absent, with reflectance dropping by almost half to 25% in the 750–780 nm range. The yellow, blue, and cyan spectra also correspond to desiccated tissue surrounding disease foci. Here, the first peak falls within 500–700 nm, with reflectance values of 22% (yellow), 29% (blue), and 31% (cyan). Second-peak values, recorded in the 700–780 nm range, are nearly identical across all three curves at approximately 22.5%. The green curve corresponds to dehydrated tissue, with reflectance ranging from 10% (first peak, 500–700 nm) to 20% (second peak, 700–780 nm).

In the loading plot (Figure 5), the first principal component p[1] explains 72.4% of total variance and exhibits a smooth, wave-like shape with positive values in the 524–728 nm range, transitioning to negative values in the NIR. This component describes the overall leaf reflectance level and disease-induced changes. The second and third components show pronounced extrema in the 524–601 nm and 702–778 nm ranges. The presence of positive and negative peaks indicates contrasting changes in pigment composition and a red-edge shift, serving as indicators of plant stress. The strong reduction in reflectance observed within lesion areas is associated with extensive necrosis and degradation of leaf tissues. The resulting spectral pattern is characteristic of diseases that induce localised tissue death and photosynthetic dysfunction.

#### 2.1.6. Spot Blotch (*Bipolaris sorokiniana*)

Figure 15 shows a sample infected with spot blotch. The red curve represents diseased areas, displayed primarily as blue zones with reduced intensity in the hyperspectral image. This curve lacks pronounced troughs, with reflectance ranging from 5% to 10%. The green spectrum corresponds to desiccated leaf tissue (first peak: 10% in 550–700 nm; second peak: 25% in 700–780 nm). The yellow curve represents tissues that were actively drying during imaging (first peak: 28% in 500–700 nm; second peak: 25% in 700–780 nm). The remaining spectra reflect spike areas desiccated due to pathogen damage. Key characteristics include: first peak in the 500–750 nm range with reflectance of 40% (blue curve) and 50% (cyan and purple curves); second-peak values recorded at 750–780 nm with identical reflectance of 30% across all three remaining curves (blue, cyan, and purple).

In Figure 6, the first principal component p[1] explains 75.3% of total variance and has a smooth, wave-like profile with positive values in the 498–728 nm range, transitioning to negative values in the NIR. The second and third components display pronounced extrema in the 498–601 nm and 702–803 nm ranges. Sharp positive and negative peaks indicate these components’ sensitivity to red-edge shifts and pigment composition changes. Spot blotch is characterised by necrosis formation and photosynthetic apparatus degradation, reducing red-region absorption and altering the NIR transition shape.

#### 2.1.7. Common Root Rot (*Bipolaris sorokiniana*)

Figure 16 shows a barley sample infected with common root rot. The red spectrum represents diseased areas, corresponding to blue zones in the hyperspectral image, characterised by low reflectance (8–10%) and the absence of distinct peaks or a red edge. The green, yellow, and purple curves correspond to healthy leaf tissue. Their first peaks lie within 500–700 nm, with reflectance values of 24% (green), 35% (yellow), and 40% (purple). The second peak, predominantly in the 700–780 nm range, shows reflectance of 20% (green), 30% (yellow), and 35% (purple). The cyan and purple spectra represent the basal stem area, with a first peak at 500–750 nm reaching 55% (cyan) and 65% (purple) reflectance. Their second peak is weakly expressed in the 750–780 nm range, with reflectance of 30% for both curves.

In Figure 7, the first principal component p[1] explains 79% of total variance and exhibits a smooth, broadband character with positive maxima, particularly in the 500–800 nm region, gradually declining toward the NIR. The second and third components display a pronounced wave-like structure with alternating positive and negative extrema. Positive values occur in the 500–600 nm and 725–800 nm ranges, while negative peaks appear predominantly in the red region (670–690 nm); subsequent positive values in the NIR range reflect the contrast between absorption zones and internal tissue scattering. The higher variability observed in healthy tissues may be related to differences in plant developmental stage and the indirect manifestation of root infections in above-ground plant organs.

#### 2.1.8. General Spectral Patterns of Disease Response

**Figure 17:**
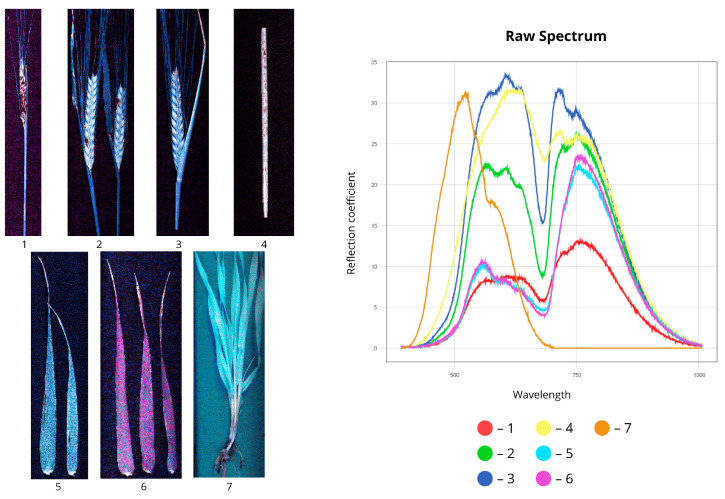
Visualisation of disease-related spectral features of barley using pseudo-RGB images: 1—Loose smut (*Ustilago nuda*), 2—Head blight (*Bipolaris sorokiniana*), 3—FHB (*Fusarium* spp.), 4—Stem rust (*Puccinia graminis*), 5—Net blotch (*Pyrenophora teres*), 6—Spot blotch (*Bipolaris sorokiniana*), 7—Common root rot (*Bipolaris sorokiniana*).

In the combined plot (Figure 17), curves exhibit two distinct peaks: the first in the 500–700 nm range and the second within 700–780 nm. Overall, most plants studied display a bimodal spectral curve structure, indicating the stability of the spectral response in barley samples regardless of disease type.

The first sample, severely affected by loose smut, shows the lowest reflectance among all samples (7–13%). The fifth and sixth samples, affected by multiple blotch diseases, resemble the first by having a more pronounced second peak in the IR range. Conversely, the *Fusarium*-infected sample exhibits the highest reflectance (32–34%). The final sample, affected by common root rot in the basal plant part, displays a spectral curve distinct from the others, with a peak in the 400–600 nm region. These results are consistent with the spectral ranges previously identified by the principal components as characteristic of barley disease manifestation.

Collectively, these findings indicate that barley diseases can be grouped into two major spectral response types: diseases associated with tissue darkening and necrosis, resulting in reduced reflectance, and diseases associated with bleaching symptoms, resulting in increased reflectance. This vital distinction provides a biologically meaningful basis for disease differentiation using hyperspectral data.

### 2.2. Quantitative Characterisation of Spectral Responses: Statistical Analysis

Analysis of the reflectance properties of barley samples infected with various diseases reveals distinct patterns for various crop diseases and tissue physiological states. Among the general patterns, diseased areas consistently exhibit minimal reflectance values within the 5–10% range, which can be regarded as a robust diagnostic trait. Maximum reflectance values are predominantly observed in desiccated tissues, with characteristic reflectance between 50% and 60%, corresponding to minimal absorption of incident light and reflecting structural degradation as well as low water balance. Meanwhile, healthy tissues generally exhibit intermediate reflectance values with low variability across samples, regardless of the disease (Table 4).

**Figure 18:**
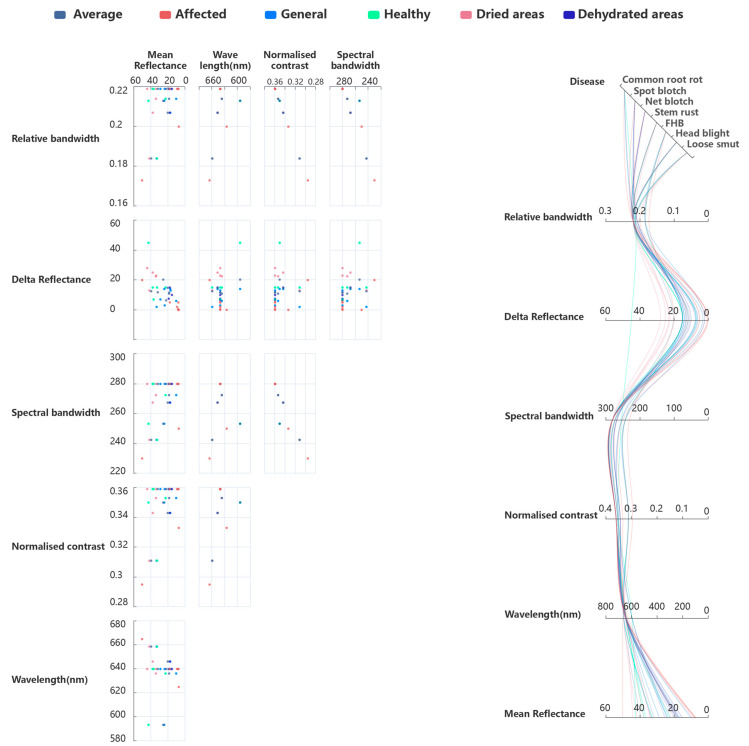
Statistical parameters of barley spectral reflectance.

Samples infected with loose smut display a sharp contrast between healthy and diseased areas, with mean reflectance (*μ*) of 22.5% and 7%, respectively. The near-zero variance values for diseased areas may serve as an indicator of spectral homogeneity under complete tissue destruction. The substantial range (∆R) between areas of different condition (up to 22.5%) points to a pronounced degradation gradient. In samples affected by head blight, overall plant spectral stability is observed, with reflectance of 22–22.5% and a coefficient of variation (CV) of 1.62%. At the same time, a strong difference in reflectance is observed between healthy and diseased areas: *μ* = 37.5% and 7.5%, respectively. Reduced reflectance through dehydrated tissue areas, corresponding to intermediate values, indicates gradual degradation. Stem rust infection causes a moderate decrease in reflectance (*μ* = 17.5%) and high variability in desiccated zones (CV = 8.08%), resulting in a smoother spectral profile compared with other barley diseases studied. Blotch diseases share similar manifestation patterns, with a maximum variation values of approximately 10%. These diseases also exhibit high spatial heterogeneity typical of blotch infections while maintaining consistently low reflectance values (7–8%). For common root rot, healthy areas exhibit the highest reflectance variability (CV = 13.45%). This likely reflects the earlier developmental stage of the crop and active disease manifestation predominantly during the tillering phase. Diseased areas show consistently low reflectance (*μ* = 9%), while the maximum reflectance range of healthy areas varies substantially (from 20% to 65%), attributable to peak photosynthetic activity and chlorophyll predominance during the intensive leaf mass accumulation stage. Distinctive disease manifestation characteristics were noted for FHB, where μ for diseased areas reaches 50%, representing an inverted spectral pattern. The high rate of change (*R* = 14.10%) compared with other diseases indicates pronounced signs of spectral heterogeneity (Table 4, Figure 18).

Analysis of disease-related spectral ranges (Table 5, Figure 18) revealed substantial similarity across most parameters, alongside several characteristic deviations. Typical disease manifestation patterns include a dominant range within 500–780 nm, a spectral bandwidth of 280 nm, and a contrast ratio of 1.42–1.56. The relative spectral bandwidth for diseased tissues is predominantly 0.219, while normalised contrast equals 0.333–0.359. However, certain diseases exhibit atypical deviations. For instance, an inverted manifestation pattern is characterised by a narrowed spectral response range (550–780 nm). This was identified primarily for FHB and dehydrated spot blotch areas, which showed a reduced spectral bandwidth of 230 nm, a contrast ratio of 1.42, and a normalised contrast of 0.295. The whole-plant sample affected by common root rot shows a slight shift toward the shortwave region (400–600 nm), which may also be related to the early developmental stage of the crop and the biochemical processes occurring in tissue cells at this phase.

Thus, the results statistically substantiate the potential of spectral methods for diagnosing barley diseases and developing automated monitoring systems. However, optimising classification accuracy requires a deep understanding of pathogen-specific spectral signatures and their distinct pathogenic mechanisms.

### 2.3. Machine Learning Approaches for Classification Modeling

A total of 5121 barley sample segments representing the studied diseases were used to develop classification models with machine learning algorithms. The distribution of segments across disease classes was uneven due to natural differences in lesion size, spatial infection structure, and lesion mosaicity; specifically, grid-based region-of-interest segmentation naturally yielded more segments in areas with a higher density of individual lesions.

Table 6 presents metrics reflecting recognition accuracy and generalisation ability of the algorithms: Macro Accuracy (R^2^Y), Cross-Validation Macro Accuracy (Q^2^Y), Log Loss, Log Loss Reduction, Micro Accuracy, and Macro Accuracy Test. Analysing these metrics collectively helps identify not only the most accurate algorithm but also the most resistant to overfitting. The R^2^Y metric characterises the proportion of explained variance in the target variable on the training set and reflects the model’s ability to approximate the original data. The highest R^2^Y values were achieved by SIMCA (0.84660) and Random Forest (0.81112). While high R^2^Y indicates good model fit, predictive capability must be assessed through validation. The more informative metric Q^2^Y reflects model stability under cross-validation. High Q^2^Y values were again observed for SIMCA (0.83969) and Random Forest (0.78784), indicating strong generalisation ability. However, unlike SIMCA, Random Forest successfully confirmed its robustness on the independent test set. For multiclass classification tasks, Log Loss—which evaluates the quality of probabilistic model predictions—is particularly relevant. The lowest Log Loss was observed for Random Forest (0.42022), indicating high confidence and well-calibrated predicted probabilities. In contrast, SVM reached a Log Loss of 0.56442, suggesting a less reliable probabilistic interpretation. Micro Accuracy, which accounts for the proportion of correctly classified instances across all classes, was highest for Random Forest (0.85178). This demonstrates strong performance even under the class imbalance typical of phytopathological diagnostics. Random Forest also achieved the highest Macro Accuracy Test value (0.84644), reflecting superior average per-class accuracy on the test set and confirming its ability to generalise to unseen data. Other algorithms yielded lower values across these metrics, with SVM exhibiting the weakest overall performance. Notably, the final four metrics are absent for SIMCA because this method is not a conventional classifier; rather, it operates via class modeling (PCA), meaning its underlying logic differs fundamentally from the other evaluated algorithms.

**Figure 19:**
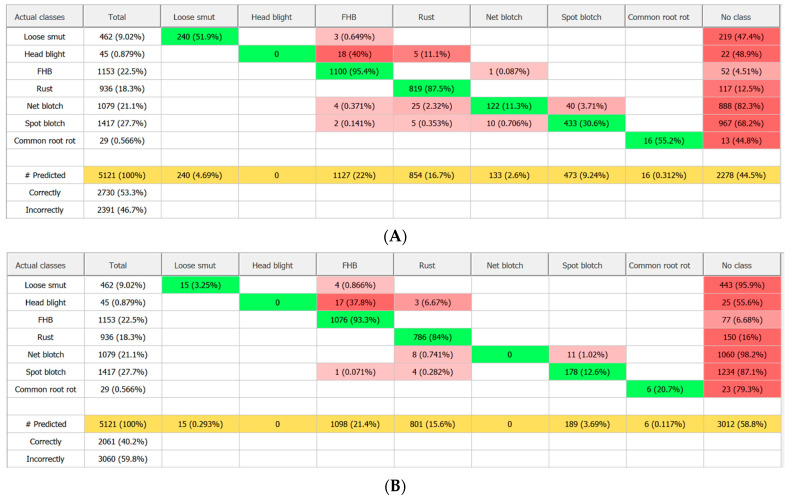

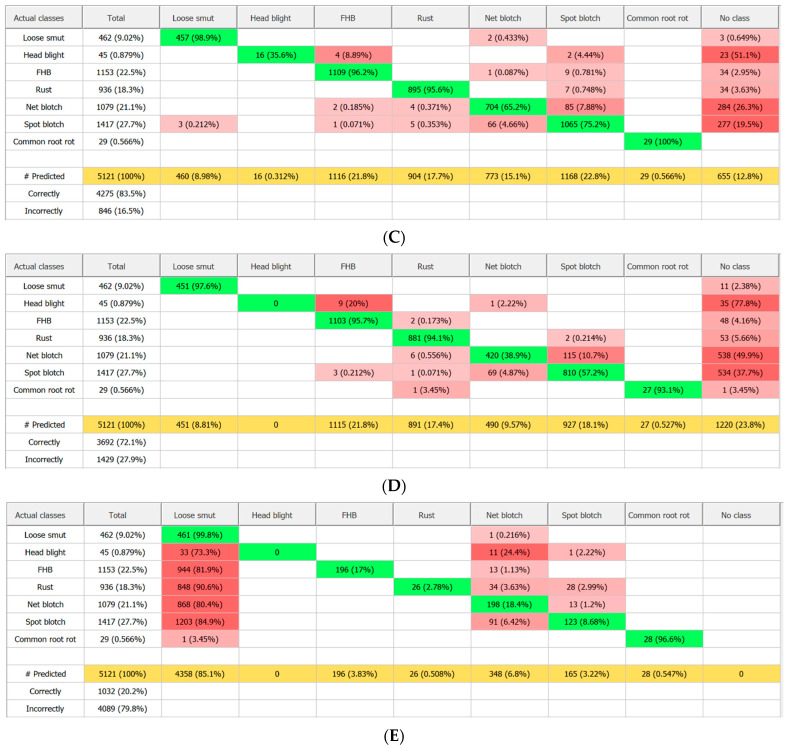
Confusion matrix ((**A**)—Neural Network (AP), (**B**)—SVM, (**C**)—Random Forest (Fast Forest), (**D**)—Maximum Entropy (SDCA), (**E**)—SIMCA). Colour coding: green—correctly classified samples; bright red—a high proportion of misclassified samples; pale red—a small proportion of misclassified samples; yellow—the total number of predicted samples.

For comprehensive model quality assessment, a set of metrics was applied, including Precision, Recall, F1-score, Binary Accuracy, Balanced Accuracy, and Matthews Correlation Coefficient (MCC), which evaluate classification performance and robustness to class imbalance based on the aggregated confusion matrix for each algorithm (Figure 19).

According to Table 7, Neural Network shows high Precision values (up to 100%) but is characterised by low Recall for several diseases, such as net blotch (11.30%) and spot blotch (30.60%), indicating a high proportion of missed instances. This combination of metrics points to a conservative classification strategy, where the model makes predictions only under high confidence, ignoring less pronounced disease cases, thereby reducing the F1-score and limiting practical applicability. SVM demonstrates pronounced limitations: while exhibiting high Precision, it shows extremely low Recall values, down to 0% for certain diseases. The model’s inability to recognise individual classes stems from SVM’s high sensitivity to kernel parameters and the difficulty of class separation under nonlinearly overlapping spectral features. As a result, the model produces imbalanced classification results. Maximum Entropy shows more balanced results but also encounters zero recognition for certain classes, e.g., head blight, and reduced Recall for diseases with poorly differentiable spectral features, such as net blotch (38.90%). The SIMCA algorithm exhibits unstable results, with high Recall accompanied by low Precision, or vice versa. For some classes, a high proportion of false-positive or false-negative classifications is observed, leading to reduced F1-scores. The method demonstrates weak discriminatory ability due to overlapping spectral characteristics of the diseases.

**Figure 20:**
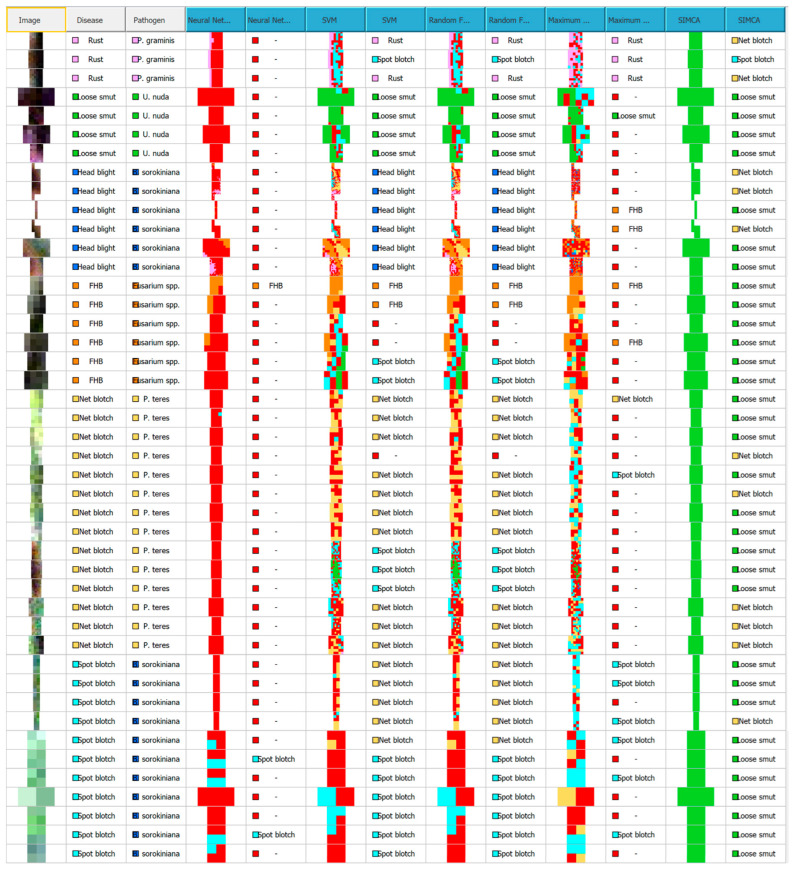
Visualisation of multiclass classification results of phytopathological barley tissue infections based on machine learning algorithms.

Random Forest delivers the best results across all metrics, as is also evident from the visual comparison of the obtained outcomes (Figure 20).

**Figure 21:**
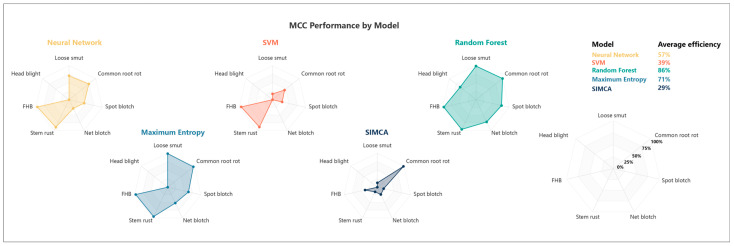
MCC-based evaluation of classification algorithms.

For most diseases, Precision and Recall values fall within the 90–100% range. For instance, loose smut Precision is 99.30% and Recall 98.90%; for stem rust, 99.00% and 95.60%, respectively; and for common root rot, metrics reach 100% across all indicators. High F1-scores of up to 97–100% demonstrate model robustness even when classifying challenging diseases such as net blotch and spot blotch, where F1-scores remain between 76.00% and 82.40%. High classification reliability even under data imbalance is shown by MCC values of 0.99–1.00. For blotch diseases, which are difficult to differentiate, MCC remains high at 0.72–0.77 (Figure 21). Balanced Accuracy exceeds 97–99% for most diseases and remains high for blotch diseases at 81.75–86.21%, indicating balanced classification.

Thus, the results confirm the high effectiveness of machine learning models for barley disease diagnosis using spectral data, with the Random Forest algorithm demonstrating particular robustness in identifying and differentiating pathogen-infected areas under high inter-class variability.

## 3. Discussion

### 3.1. Interpretation and Diagnostic Relevance of Disease-Specific Spectral Signatures in Barley

The operating principle of HSI lies in the selective capture of specific plant pixels and their conversion into spectral information, reflecting the biochemical composition and physiological state of individual plant tissues and the whole plant. The VNIR range (400–1000 nm) is optimal for plant disease identification. Principal component analysis revealed the spectral ranges contributing most to the variability of reflectance characteristics in infected tissues. Based on the factor loadings, the most informative spectral region spans 550–700 nm and reflects pigment type and absorption characteristics. This range encompasses the green spectral region (~520–560 nm), the chlorophyll absorption red zone (~650–680 nm), and the red-edge region (~700 nm). The second component primarily captures the 700–800 nm spectral region associated with plant structural features. The most significant wavelengths for diagnosing barley condition are ~600 nm, ~700 nm, and ~750 nm.

Spectral characteristics differ among diseased, dehydrated, desiccated, and healthy barley tissues. Healthy tissues exhibit high reflectance in the green and NIR regions, a pronounced minimum in the red zone, and a steep rise in the red-edge region. The steep red-edge rise indicates preserved cell wall structure and high pigment content. Spectral curves of healthy plant areas display two distinct peaks (500–700 nm and 700–780 nm), separated by the red edge at 700 nm. In the VIS range, reflectance level depends on pigment composition and absorption characteristics. High reflectance originates from zones with a predominance of amyloplasts—such as the basal stem area—while lower reflectance comes from chlorophyll-containing zones, where light is absorbed for photosynthetic activity. In the NIR range, reflectance depends on the structural properties of the plant and its individual organs. Overall, healthy tissues exhibit an intermediate reflectance level. Compared to healthy tissues, dehydrated areas exhibit reduced reflectance due to pigment degradation and decreased cell turgor, which alter optical density and mesophyll structure. Curves representing diseased areas display a single pronounced peak, a smoothed red edge, and varying reflectance levels depending on the infecting pathogen. This spectral shape indicates chlorophyll breakdown and disruption of cellular structure.

Zones affected by loose smut, head blight, common root rot, stem rust, net blotch, and spot blotch are characterised by reduced reflectance due to dark-coloured pigments. The dark colouration enhances light absorption and reduces reflectance, resulting in lower spectral intensity in pathogen-affected organs. The dark pigmentation of loose smut spores may be associated with the presence of melanin-like pigments, as in *Podospora anserina* [45], *Pestalotiopsis microspora* [46], and *Ustilago maydis* [47].

Diseases such as spot blotch, head blight and common root rot are caused by fungi of the genus *Helminthosporium*, appearing as dark spots on leaves, stems, and other plant parts. The pathogens synthesise hydroxyanthraquinones (catenarin, helminthosporin, and emodin), which are produced under stress and during secondary metabolite synthesis. These molecules possess double bonds and are coloured in reddish-brown hues due to their conjugated chromophore system [48]. The same pigments are produced by the common root rot pathogen *Bipolaris sorokiniana* [49]. Such pigments exhibit toxic properties and play a role in pathogenesis, promoting tissue destruction [50] and enhancing fungal resistance to adverse conditions and survival in soil [51]. Net blotch pathogens can synthesise hydroxyanthraquinones (cynodontin, erythroglaucin, catenarin, helminthosporin, and tritisporin), characteristic of fungi of the genus *Pyrenophora*. This is an oxidised form of hydroxyanthraquinones, imparting a more saturated brown colour to lesions [51].

Stem rust, caused by the fungus *Puccinia graminis*, manifests as dark spots on barley stems, which in hyperspectral images appear as isolated blue areas. The pathogen spreads via spores containing melanin-like pigments. Disease manifestation is driven by urediniospores accumulating flavonoid derivatives and carotenoids, including β- and γ-carotene [52], which provide protection against UV radiation and oxidative processes, while also giving spores their distinctive reddish colouration. Teliospores may contain melanin-like compounds, as supported by the presence of genes encoding laccase-like proteins [53] and laccase enzymes [54] in rust pathogens. Reduced reflectance intensity caused by dark pathogen pigments, sporulation, and tissue degradation is supported by findings from other studies [54]. The dark colouration of pathogens may result from adaptation, ensuring effective dispersal and spore viability under agricultural agroecosystem conditions.

In response to infection, plants activate defense mechanisms accompanied by enhanced formation of melanin and various phenolic metabolites, manifested as visual darkening of infected tissues [55]. Dark pigments in spore structures serve a protective function [56]. They absorb ultraviolet and part of visible radiation, having adapted to open agroecosystem conditions where spores are subjected to intense insolation. Melanised cell walls exhibit greater density and mechanical strength, increasing spore resistance to desiccation, temperature fluctuations, and oxidative agents. Cell wall reinforcement facilitates penetration into plant tissues. Thus, dark pigments contribute to the long-term persistence of the pathogen in soil and on plant residues.

Owing to their absorption capacity, diseased areas can be differentiated by reduced reflectance and a smoothed red-edge curve. Diseases characterised by white coating and tissue lightening appear in hyperspectral images in red or orange, with higher light reflectance intensity against the background of healthy tissue; isolated areas and greater variability in light representation are visible in the image. FHB manifests as a pale pink coating and desiccation of barley spikes. It is caused by fungi of the genus *Fusarium*, which produce polyketide pigments (aurofusarin, bikaverin, rubrofusarin, and neurosporaxanthin) [57,58] that weakly colour the spores. The absence of intensely coloured pigments leads to strong scattering of incident light. The white coating is characterised by elevated reflectance across a broad visible spectral range, owing to the lack of light absorption by pigments. This colouration also serves adaptive functions. The mass production of light, dry, colourless spores facilitates wind dispersal. The white coating reflects light, blending with the natural reflective structures of the leaf, complicating early infection detection, reducing overheating of surface mycelium, and protecting cells from photodestruction.

The inverted spectral behaviour observed in FHB differs fundamentally from that of diseases associated with dark pigmentation and necrotic lesions. In addition to the reflective properties of the fungal mycelium, FHB infection is accompanied by bleaching and desiccation of spike tissues, resulting in substantial chlorophyll loss and reduced absorption in the red region of the spectrum. The degradation of photosynthetically active pigments decreases the depth of the characteristic reflectance minimum near 670 nm, while the formation of dry surface structures increases diffuse scattering throughout the visible range. Consequently, the reflectance intensity of FHB-infected tissues exceeds that of both healthy tissues and dark-pigmented lesions, producing the atypical spectral pattern observed in this study. The high variability recorded for FHB is likely associated with heterogeneous disease development within individual spikes, where healthy, partially infected, bleached, and desiccated tissues may coexist, and contribute differently to the overall spectral response.

**Figure 22:**
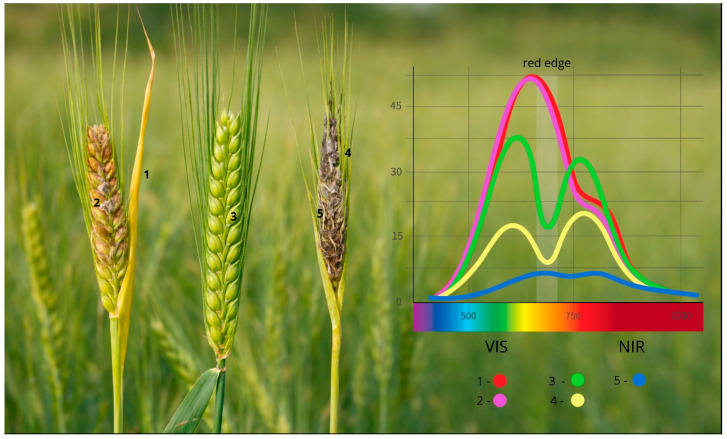
Comparative spectral characteristics of healthy barley and phytopathological conditions (1—Desiccated areas, 2—*Fusarium* infection, 3—Healthy plant, 4—Dehydrated areas, 5—Loose smut infection).

Lightening due to tissue desiccation and chlorophyll loss alters optical properties; light scattering increases with reduced absorption, especially in the visible spectrum, raising reflectance intensity. In hyperspectral images, such areas appear as isolated or discrete zones near the disease focus or at leaf tips. As seen in the comparative summary plot (Figure 22), the reflectance of diseased barley varies depending on pathogen type. *Fusarium* infections with white coating increase overall reflectance intensity, whereas loose smut infections with extensive darkening reduce reflectance.

Disease development is accompanied by chloroplast degradation and a reduction in chlorophyll concentration, which is one of the earliest physiological manifestations of biotic stress. Phytopathogenic fungi produce toxins and enzymes that damage cell membranes and disrupt photosynthetic processes. As a result, light absorption in the red region of the spectrum decreases, the characteristic reflectance minimum becomes less pronounced, and the red edge is shifted or reduced in intensity. Such changes are considered indicators of impairment of the photosynthetic apparatus in plants. As the infection progresses, chlorophyll content decreases more rapidly than carotenoid concentration. This alters the pigment balance and leads to the appearance of yellowish or brownish tissue discolouration. Carotenoids perform an antioxidant function and protect the photosynthetic apparatus from excessive accumulation of reactive oxygen species; however, under severe infection, their protective capacity becomes insufficient. Degradation of the pigment complex is therefore accompanied by reduced photosynthetic efficiency and changes in spectral characteristics within the visible range. Infection also triggers a range of plant defense responses, including the accumulation of phenolic compounds, lignin, phytoalexins, and oxidation products of polyphenols. These substances contribute to the formation of mechanical and chemical barriers that restrict pathogen spread. Oxidation of phenolic compounds by polyphenol oxidases and peroxidases results in the formation of dark-coloured polymers, which are visually expressed as brown and necrotic lesions. Damage to vascular tissues, disruption of transpiration, and degradation of cell membranes lead to loss of turgor and tissue dehydration. Reduced water content alters the refractive properties of cellular structures and decreases light scattering within the mesophyll. Consequently, changes in reflectance are observed not only in the visible spectrum but also in the near-infrared region. Thus, the physiological condition of plants has a direct influence on their spectral characteristics. The shape of spectral curves and their reflectance values can serve as reliable indicators for diagnosing infected plants and identifying the type of disease.

### 3.2. Statistical Interpretation of Spectral Response Characteristics

The spectral characteristics obtained enable the identification of fundamental biophysical changes occurring in plant tissues. Interpreting the statistical indicators from a physiological perspective reveals that the reduction in reflectance to 5–10% is primarily associated with the breakdown of cellular structure, increased light absorption due to chlorophyll and other pigment degradation, and the accumulation of necrotic tissues. The rise in reflectance in desiccated areas, reaching up to 60%, is mainly driven by reduced cellular water content, increased light scattering by disrupted cell structures, and the consequent change in refractive index. Examination of barley disease features with diagnostic potential shows that loose smut and head blight follow a classical classification model, where reflectance values decline from healthy tissues to necrotic, pathogen-infected zones. FHB, in contrast, exhibits an atypical pattern, where higher reflectance is observed in infected areas. This may be associated with microstructural-level disruptions leading to increased scattering, as well as the high reflectivity of fungal mycelium. Blotch diseases require spatial analysis due to the relative complexity of their classification, arising from the mosaic nature of infection, as confirmed by their high coefficients of variation. Furthermore, the high variability of healthy tissues during the crop tillering stage, driven by active photosynthetic processes, may occasionally reduce diagnostic contrast, thereby necessitating additional spectral indices. Thus, based on reflectance characteristics, diseases can be broadly divided into classical (with reduced reflectivity) and inverted (FHB).

The universality of the 500–780 nm spectral range indicates the preservation of the basic spectral structure across most diseases. Narrowing of the range to 550–780 nm in FHB indicates the loss of the blue region, primarily due to reduced photosynthetic activity, partial chlorophyll degradation, and the consequent loss of sensitivity in the shortwave portion. This is also particularly evident in blotch diseases at intermediate stages. The shift of the spectral range toward 400–600 nm in common root rot, observed during whole-sample analysis, is associated with suppressed reflectance in the NIR region, disrupted pigment composition, and an increased contribution of “defensive” compounds such as phenolics and carotenoids. Conditions favourable for diagnostics are characterised by higher contrast values (1.56), enabling clearer separation of spectral responses. Reduced contrast in certain diseases (FHB) or in specific tissue areas (dehydrated areas in spot blotch) indicates overlapping spectral characteristics, which may affect diagnostic sensitivity; however, these minor variations do not significantly impact the results. Diseases with high variability generally exhibit a narrowing of the spectral range in which pathogenesis features manifest.

Thus, spectral characteristics allow a fairly clear separation of diseases by their mechanism of damage into necrotic, structural-degradative, and dehydrative types. The main challenges in diagnosis include high variability in statistical indicators, reduced contrast, and non-standard spectral patterns. The identified spectral patterns reflect not only the presence of disease but also its stage of development, the degree of tissue damage, and the intensity of physiological stress in the plant.

### 3.3. Interpretation of Algorithmic Behavior in Machine Learning Classification

The advantage of the Random Forest algorithm lies in its construction of an ensemble of multiple decision trees, each trained on a random subsample of the original data and a feature subset, reaching a final decision through aggregation. This substantially reduces the risk of overfitting by collectively decreasing correlation among trees and enhancing robustness to noise. The final decision is made by aggregating the voting results of the trees and averaging the probability values.

Recent advances in multimodal artificial intelligence for precision agriculture further demonstrate that combining heterogeneous data sources (e.g., spectral, spatial, and contextual information) significantly improves disease detection performance and robustness of classification models under field variability conditions [59].

Moreover, Random Forest effectively handles high-dimensional hyperspectral data comprising several hundred spectral channels by automatically selecting the most informative features and mitigating multicollinearity, including that between adjacent spectral channels. Unlike SVM and neural networks, this algorithm does not require complex hyperparameter tuning and can effectively model intricate nonlinear relationships among features. It also demonstrates robustness to class imbalance, achieving higher Recall values for diseases with limited training samples—a critical factor in the classification of rare diseases. An equally important advantage of Random Forest is its resilience to local classification uncertainty, particularly in cases involving dehydrated or mixed lesions, where Maximum Entropy and SIMCA methods fall short. For the latter, component selection and training set homogeneity play crucial roles, whereas for Random Forest, capturing the components with the greatest informativeness is paramount.

Despite Random Forest’s high effectiveness, a decline in accuracy, F1-score, and MCC is observed for blotch diseases. This is attributable to their physiological similarities to necrosis and chlorosis, leading to overlapping spectral signatures, especially in pigment-related and leaf-structure-sensitive wavelength ranges. Such overlapping information can hinder unambiguous classification of infected areas, particularly when lesion zones exhibit mixed symptoms or partial tissue dehydration.

An additional factor contributing to spectral overlap and classification confusion among head blight, spot blotch, and common root rot is their shared aetiology. These diseases are caused by *Bipolaris sorokiniana* and therefore induce similar physiological and biochemical changes in plant tissues, including pigment degradation, necrosis development, and accumulation of pathogen-derived metabolites. As a result, their spectral signatures partially overlap, reducing class separability and increasing the likelihood of misclassification. This finding suggests that practical deployment of hyperspectral disease classification systems may benefit from integrating spectral information with disease localisation on specific plant organs and additional contextual features.

Thus, the Random Forest algorithm combines high accuracy and reliability with flexibility in application to high-dimensional hyperspectral data while accounting for noise effects, making this method more universally applicable to the variable and complex spectral signals of biological objects. Despite the class imbalance limitation in the dataset, the proposed algorithm demonstrated strong performance on the trained model. On the other hand, class imbalance may reflect real field conditions, where the distribution of healthy and diseased plants is inherently uneven.

## 4. Methods

### 4.1. Study Objects and Plant Sampling Sites

The study focused on samples of common barley (*Hordeum vulgare* L., 1753) infected with fungal pathogens: *Ustilago nuda* ((Jensen) Rostr., 1889; causal agent of Loose smut), *Bipolaris sorokiniana* (Sacc., 1890; causal agent of Head blight, Spot blotch, Common root rot), *Fusarium* spp. (Link, 1809; causal agent of FHB), *Puccinia graminis* (Pers., 1797; causal agent of Stem rust), *Pyrenophora teres* (Drechsler, 1923; causal agent of Net blotch).

Sampling was conducted in the main grain-producing districts of northeastern Kazakhstan (Pavlodar region) during the crop growing season. The sample set was formed by collecting infected plants at key phenological stages of barley development (BBCH 25–35, 39–59, 61–75). Growth stages were recorded according to the Biologische Bundesanstalt, Bundessortenamt und Chemische Industrie (BBCH) scale. The indicated barley developmental stages represent the periods during which diseased plants were collected in the field; however, not all diseases were represented at each stage, as disease occurrence reflected their natural development during the growing season.

The sampling area is characterised by typical steppe agricultural landscapes, with flat terrain and a sharply continental climate. The growing season features moderately warm weather and uneven precipitation: mean air temperatures ranged from +13 to +22 °C; precipitation at the beginning of the growing season was within 15–22 mm, rising to 65–70 mm in the latter half. Soils are medium-loamy chestnut and dark chestnut types (calcareous composition, slightly alkaline reaction, pH 7.3–7.6, organic matter content 2.5–3.0%). Prevailing winds are northeasterly and southwesterly at 3–5 m/s. Productive moisture reserves (0–50 cm soil layer) were 60–80 mm at the start of the growing season, 30–40 mm in June, and 70–90 mm in late July.

Local sampling sites were selected based on distal multispectral sensing using a DJI Mavic 3M (SZ DJI Technology Co., Ltd., Shenzhen, China). Multispectral camera specifications: green (G): 560 ± 16 nm; red (R): 650 ± 16 nm; red edge (RE): 730 ± 16 nm; near-infrared (NIR): 860 ± 26 nm. Image sensor: 1/2.8-inch CMOS. Lens: FOV 73.91° (61.2° × 48.10°), equivalent focal length 25 mm, aperture f/2.0. Electronic shutter speed: 1/30–1/12,800 s. To identify stress zones where disease-affected plants were presumed to predominate, vegetation index maps were generated using MCARI (Modified Chlorophyll Absorption Ratio Index) (1) [60] and NDRE (Normalised Difference Red Edge Index) (2) [61], calculated as follows [62]:

(1) $$MCARI=\left[\left(R_{700}-R_{670}\right)-0.2(R_{700}-R_{550})\right]\times \frac{R_{700}}{R_{670}},$$

where *R*_550_, *R*_670_, and *R*_700_ are the reflectance values at wavelengths of 550 nm, 670 nm, and 700 nm, respectively.

(2) $$NDRE=\frac{NIR-RedEdge}{NIR+RedEdge}$$

where NIR is the reflectance in the near-infrared range; Red Edge is the reflectance in the red-edge spectral region.

**Figure 23:**
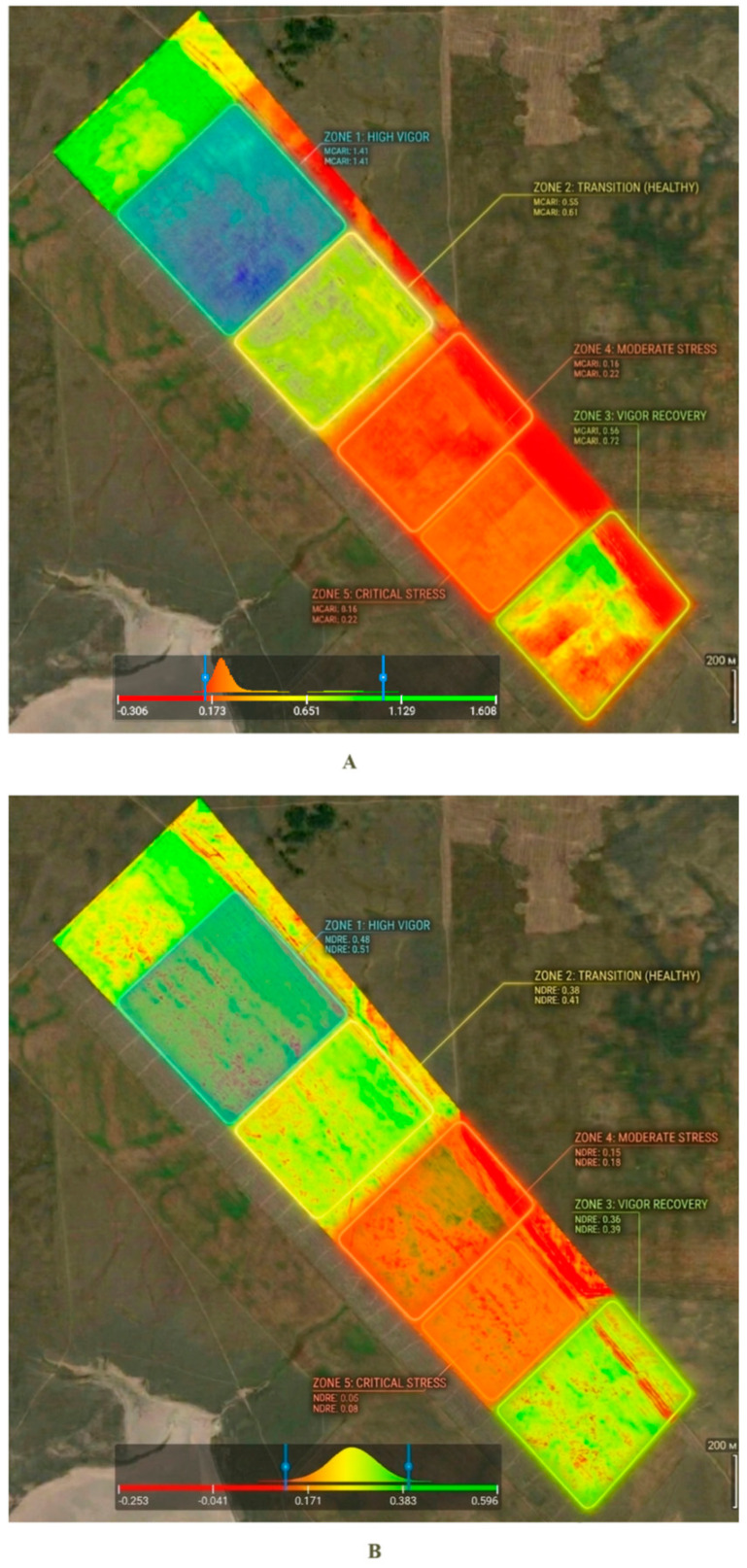
Spatial differentiation of barley crop infection zones based on multispectral remote sensing data: (**A**) MCARI index; (**B**) NDRE index.

The MCARI index is highly sensitive to changes in chlorophyll content in upper leaves. Since most diseases begin their development and manifestation with the destruction of chloroplasts, this index enables the detection of plant energy loss, particularly at early stages. The NDRE index indicates overall stress status and penetrates into the middle canopy layer, where disease development often begins; even under dense biomass, it continues to detect subtle differences in crop health. Integrating these indices provides a more comprehensive picture, as overlapping stress zones within a specific area substantially increase the likelihood of correctly identifying an ongoing pathological process. Soil noise is mitigated by the MCARI index, which resolves data at higher detail even in sparse stands (typically resulting from partial biomass loss) affected by pathogens. An MCARI value below 0.2 indicates the onset of irreversible degradation processes in plant cells. An NDRE value of 0.3 serves as a threshold: values above this indicate sufficient nitrogen and leaf mass, whereas lower values suggest disease symptoms. Accordingly, as shown in Figure 23, zones 4 and 5 were selected, as they clearly demonstrate a critical decline in these indicators, making these areas priorities for more detailed proximal analysis and sample collection for bio-examination. The selection of these zones is further supported by the interpretation of index values on the colour scales, where the right side of the “slope” corresponds to healthy plant mass, while the left “tail” relates to statistical anomalies deviating from the overall healthy field matrix. The selected zones capture the transitional areas, from the initial decline in chlorophyll levels to structural vegetation degradation and entry into the “epicenter”.

A total of 263 barley samples were collected: loose smut—32, head blight—36, FHB—27, stem rust—38, net blotch—41, spot blotch—44, common root rot—45. These samples were used for hyperspectral processing and construction of the working dataset. Samples collected in the field were transported to the laboratory and used for hyperspectral imaging, processing, generation of datasets for machine learning-based analysis, and classification model development.

### 4.2. Assessment of Phytopathogen Infection in Barley

Sampling was conducted to form a representative set of pathogen-infected plants. Plant collection within the selected areas followed an interval of 25–50 paces, progressing 25–50 m from the field edge and 200–300 m into the crop stand. Uniform infection distribution warranted a triangular or rectangular sampling pattern, while focal infection patterns necessitated diagonal or checkerboard transects [63]. Plants exhibiting characteristic disease symptoms were collected for the following diseases: Loose smut (*Ustilago nuda*), Head blight (*Bipolaris sorokiniana*), FHB (*Fusarium* spp.), Stem rust (*Puccinia graminis*), Net blotch (*Pyrenophora teres*), Spot blotch (*Bipolaris sorokiniana*), Common root rot (*Bipolaris sorokiniana*). Phytopathogens were diagnosed using laboratory methods (pure culture isolation, microscopy) in accordance with State Standard 12044–93 “Methods for Determining Disease Contamination” [64]. Disease severity was assessed using the CIMMYT visual scale (0–100%), representing the percentage of plant area affected. Scores were categorised as 0% (no symptoms), 1–20% (mild), 21–50% (moderate), and 51–100% (severe infection). This scale is widely used for cereal disease phenotyping and ensures consistent severity assessment [65].

### 4.3. Hyperspectral Imaging

Laboratory studies were conducted at the Biological Research Laboratory of Toraighyrov University (Pavlodar, Kazakhstan).

#### 4.3.1. Preparation of Plant Samples for Spectral Imaging

To ensure appropriate conditions for hyperspectral image acquisition, calibration panels with a uniform, matte surface were prepared beforehand. Plant samples were positioned to ensure correct data processing during segmentation. Barley samples were fixed at a distance of 30–40 cm from the hyperspectral camera in their natural orientation, predominantly with infected areas facing upward. Prior to imaging, calibration was performed using reference standards, where the white reference was a fluoropolymer panel (Spectralon (99%)—Labsphere, Inc., North Sutton, NH, USA) [66], reducing noise effects on samples and ensuring high reflectance accuracy. Radiometric calibration was performed using both dark current subtraction and white reference correction. Raw digital numbers (DN) were converted into reflectance values (normalised reflectance) using the standard Equation (3) [67]:

(3) $$I_{norm}=\frac{I_{raw}-I_{dark}}{I_{white}-I_{dark}},$$

where *I*_raw_ is the measured intensity, *I*_dark_ is the intensity measured from a matte black reference panel, and *I*_white_ is the intensity measured from a Spectralon white reference panel.

#### 4.3.2. Hyperspectral Image Acquisition and Processing: Technical Parameters and Image Processing

Imaging was performed using a scanning-type VNIR (400–1000 nm) hyperspectral camera, FigSpec^®^ FS-13 (CHNSpec Technology, Hangzhou, China). The camera operates across 1200 spectral channels with a spectral resolution of 2.5 nm, employing a transmission diffraction grating and a CMOS detector. Detection of small disease lesions is enabled by a spatial resolution of 1920 pixels/line (pixel size = 5.86 µm) and a frame rate of 128 fps. To ensure reproducibility, all hyperspectral measurements were performed under controlled laboratory conditions. The illumination geometry was kept constant (nadir view with two halogen lamps positioned at 45° on opposite sides of the sample). The camera-to-sample distance was maintained in the range of 30–40 cm depending on the size of the disease lesion, while ensuring consistent field-of-view and spatial resolution. Identical camera acquisition settings were used for all measurements. Collectively, these parameters ensure high sensitivity and low noise perception for more accurate analysis of the absorption and reflectance characteristics of diseased areas and various morphological structures [68].

Raw images were loaded into Breeze software (v2024.2.0) for large-scale data processing. Hyperspectral images are three-dimensional arrays with a two-dimensional spatial structure and high spectral detail. Correction coefficients were calculated using calibration standards; to eliminate background pixel and noise effects, masks generated using ROI tools, as well as clustering algorithms and morphological filtering, were applied. The resulting masks were used for spectral export and preprocessing via smoothing, standard normal variate (SNV) normalisation, and centering to ensure the informativeness of the extracted data.

#### 4.3.3. Assessment of Plant Sample Condition Based on Spectral Analysis and PCA

Visualisation of preliminary research results was achieved by reducing the dimensionality of hypercubes and presenting them layer-by-layer as principal component images based on a PCA model. Additional information on multi-component images was obtained from Raw Spectrum and Variance Scatter plots. Raw Spectrum was used to extract spectra in the VNIR range within the defined focus areas; Variance Scatter was used to reveal pixel distribution across principal components PC1(t[1])–PC6(t[6]), with t[1] and t[2] selected as the most informative. Principal components were selected based on the cumulative explained variance criterion. Components accounting for up to 95% of total variance were retained for further analysis. Eigenvalues and scree plot analysis were used to determine the significance of each component. PC1 and PC2 were selected for visualisation and interpretation because they contained the highest proportion of spectral variance and provided the best separation between healthy and diseased tissues. Higher-order components (PC3–PC6) were excluded from visualisation due to their low signal-to-noise ratio and limited interpretability for classification purposes. PC1 primarily represented overall spectral intensity variation associated with chlorophyll content and mesophyll structure, whereas PC2 captured secondary variability linked to structural and morphological differences in infected tissues. In the Variance Scatter plot, the point cloud was formed based on the principle of grouping pixels by spectral similarity, with maximum density corresponding to red colouration.

#### 4.3.4. Machine Learning Methods for Identification and Differentiation of Barley Diseases

Machine learning algorithms were used to construct and train a model for subsequent identification and differentiation of diseased areas on barley plants through optimisation and combination of spectral characteristics. A PCA model was developed to extract and visualise principal components, followed by reference modeling for background removal and region-of-interest extraction.

Five machine learning algorithms were tested for automatic classification and differentiation of barley diseases:

- Neural Network (AP)—works with linear characteristics of the studied objects but may encounter difficulties in classification when complex feature sets are present [69];
- SVM—a method based on constructing an optimal separating boundary between classes, effective with high-dimensional data [70];
- Random Forest (Fast Forest)—an ensemble method based on multiple decision trees, providing high accuracy and noise robustness [71];
- Maximum Entropy (SDCA)—a linear model (logistic regression), trains quickly on large, sparse datasets, suitable for multiclass tasks [72];
- SIMCA—operates on a principal component basis, modeling each class separately and assessing class membership by distance [73].

Feature selection was performed using a combination of PCA-based dimensionality reduction and variance thresholding. Spectral bands with low variance (<1%) and high inter-band correlation (>0.95) were excluded to reduce redundancy and computational complexity. All machine learning models within the pipeline were implemented using the Breeze library with default (built-in) hyperparameter settings. Model-specific parameters were not tuned and were used as provided by the library. Model performance was evaluated using stratified k-fold cross-validation (k = 5), ensuring balanced representation of all disease classes in each fold. This approach minimised overfitting and improved generalisation ability of the models.

**Figure 24:**
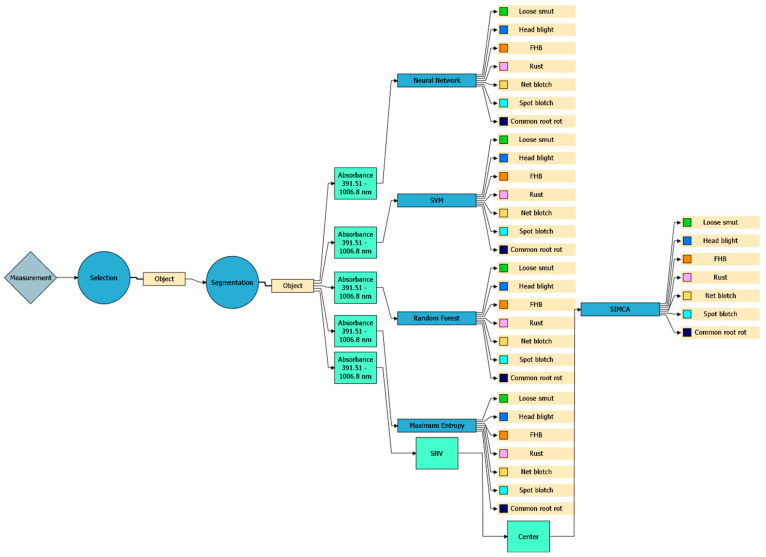
Workflow of machine learning models: from data acquisition (raw input data) to the classification stage.

The schematic workflow, from loading raw input data into the software to the classification stage, is presented in Figure 24. Cross-validation was employed during training; a manual region-of-interest selection mode was applied, with subsequent grid-based labeling for precise delineation of individual lesion areas and their accurate identification and differentiation. A lesion-guided ROI-based patch extraction strategy was employed, where hyperspectral patches were generated exclusively from annotated diseased regions. The dataset comprised 5121 hyperspectral patches derived from 263 barley plant samples. For machine learning model development, the dataset was split into training (70%) and testing (30%) subsets at the ROI level to prevent data leakage between subsets, ensuring class balance across all partitions. This ensured that all patches from a given ROI were assigned exclusively to a single subset. However, it should be noted that neither the training nor the test datasets provide a fully independent external validation setting. As a result, the reported performance reflects model generalisation under controlled experimental and local agroecological conditions.

The dataset exhibited a moderate class imbalance, with the number of plant samples per disease category ranging from 27 (FHB) to 45 (common root rot). The selection of the number of samples was also guided by the severity of disease infection and the spatial extent of infection foci. Accordingly, disease categories characterised by smaller and fewer infection foci were represented by a larger number of samples to ensure sufficient variability and coverage of symptom expression. To mitigate potential model bias toward majority classes, stratified sampling was applied during dataset splitting to preserve the original class distribution across training and test subsets.

In addition, class-wise performance metrics (Precision, Recall, and F1-score) were used instead of relying solely on overall accuracy, ensuring that minority classes were adequately evaluated. Balanced Accuracy and Matthews Correlation Coefficient (MCC) were also adopted to reduce the influence of class imbalance on performance interpretation. Potential bias toward dominant classes was further reduced through ensemble-based learning methods (Random Forest) and margin-based classifiers (SVM), which are less sensitive to uneven class distributions compared to simple linear models.

### 4.4. Statistical Data Processing

The spectral characteristics and classification performance metrics were evaluated using descriptive statistics and analysis of variance. The formulas for the key indicators are presented in Table 8.

The results of the statistical analysis were used to evaluate spectral patterns and the performance of classification models for the identification and differentiation of barley infections by disease pathogens.

## 5. Conclusions

This study confirms the high diagnostic value of hyperspectral imaging and machine learning algorithms for the identification and differentiation of barley phytopathologies based on spectral signatures.

Key diagnostic zones were identified: 520–560 nm (green region), 650–680 nm (chlorophyll absorption), and 700–730 nm (red edge). Healthy tissue areas are characterised by a pronounced minimum in the red zone, high reflectance values in the NIR, and a sharp rise in the red-edge region, reflecting active photosynthesis and cellular structural integrity. Pathogen-infected tissues, in contrast, exhibit red-edge smoothing due to altered mesophyll structure, pigment degradation, and accumulation of pathogenesis products. Spectral responses are intrinsically linked to the biochemical nature of the infection. Diseases with dark pigmentation (loose smut, spot blotch, stem rust) lead to reduced reflectance due to intense light absorption by phenolic and melanin-like compounds. Diseases with a whitish hue or light coating (FHB) exhibit increased reflectance due to enhanced light scattering. Tissue dehydration also increases reflectance relative to pathogen-infected areas due to reduced water content and altered cellular optical properties.

Statistical analysis distinguished two main types of spectral behaviour: the classical type, where tissue infection leads to decreased reflectance, and the inverted type, where infection is accompanied by increased reflectance (characteristic of *Fusarium*). Substantial variability in spectral data and low inter-class contrast can, however, complicate disease diagnosis, primarily posing difficulties in identifying blotch diseases or mixed infections, where overlapping spectral signatures indicate a need for further method optimisation for mixed lesions and represent a promising direction for future research.

Analysis of machine learning algorithms identified Random Forest as the most effective method for barley disease classification based on spectral data (accuracy 90.13%, MCC 0.86). Its high performance is attributed to its ability to handle high-dimensional data, resistance to noise, and low susceptibility to overfitting, providing an optimal balance of accuracy, robustness, and generalisation ability. This algorithm demonstrates high classification accuracy, particularly for diseases with pronounced spectral differences; however, its effectiveness decreases when spectral signatures overlap (blotch diseases, mixed phytopathologies).

The absence of an external validation dataset drawn from independent geographic regions, as well as the relatively small sample size, remains a limitation of this study. Although internal validation strategies, including a hold-out test set and cross-validation, were employed to ensure model reliability, further evaluation using external datasets from diverse agroecological zones is required to assess model generalisation fully. In addition, further expansion of the training dataset for each disease class would improve the representation of disease-specific spectral signatures and potentially enhance model generalisation. These limitations are commonly encountered in hyperspectral plant disease studies due to the high cost and complexity associated with acquiring standardised external datasets under comparable imaging conditions.

Practically, this approach demonstrates promising potential for precision agriculture, specifically in early disease detection and field monitoring. Future research will focus on developing automated, real-time monitoring systems suited to large-scale farming using unmanned aerial vehicles equipped with hyperspectral sensors and supported by machine learning classification models.

Thus, the results demonstrate that the integration of hyperspectral analysis, statistical evaluation of spectral features, and machine learning enables reliable diagnosis and classification of barley diseases according to infection type through the detection of latent physiological changes in plants. This represents an important step toward the development of automated early diagnostic and phytopathological monitoring systems for agroecosystems.

## Figures and Tables

**Table 1:** Variance contribution of principal components (PC1–PC6).

Barley Disease	PC1	PC2	PC3	PC4	PC5	PC6
	%					
Loose smut	65.90	12.60	1.46	0.384	0.151	0.068
Head blight	88.40	5.21	0.644	0.161	0.077	0.045
FHB	92.40	2.94	0.776	0.102	0.059	0.055
Stem rust	89.00	4.24	0.32	0.048	0.028	0.016
Net blotch	72.40	8.41	1.21	0.368	0.147	0.067
Spot blotch	75.30	7.55	1.18	0.289	0.140	0.056
Common root rot	79.00	14.40	1.43	0.358	0.122	0.058

**Table 2:** Overview of variance explained by principal components: key statistics.

Component	Explained Variance	Cumulative Variance	Eigenvalue (Total = 1)
	%		
PC1	80.34	80.34	0.8034
PC2	7.91	88.25	0.0791
PC3	1.00	89.25	0.0100
PC4	0.24	89.49	0.0024
PC5	0.10	89.59	0.0010
PC6	0.05	89.65	0.0005

**Table 3:** Factor loading and wavelength ranges (PC1 and PC2) for spectral features of barley diseases.

Component	Peak	PC1				PC2			
		Load Factor		Wavelength		Load Factor		Wavelength	
		Min	Max	Min	Max	Min	Max	Min	Max
Loose smut	1st	0.025	0.06	524	677	-	-	-	-
	2nd	0.025	0.04	702	778	0.025	0.07	702	803
Head blight	1st	0.025	0.05	524	660	-	-	-	-
	2nd	0.025	0.03	702	803	0.025	0.06	728	853
FHB	1st	0.025	0.06	524	677	-	-	-	-
	2nd	0.03	0.04	702	753	0.025	0.07	600	702
Stem rust	1st	0.025	0.06	500	652	-	-	-	-
	2nd	0.025	0.04	652	803	0.025	0.07	702	853
Net blotch	1st	0.025	0.07	524	728	0.025	0.06	524	600
	2nd	-	-	-	-	0.025	0.05	728	828
Spot blotch	1st	0.025	0.07	524	702	0.025	0.05	524	576
	2nd	-	-	-	-	0.025	0.05	728	853
Common root rot	1st	0.025	0.07	525	660	-	-	-	-
	2nd	0.025	0.07	700	825	0.025	0.07	700	850

**Table 4:** Barley reflectance characteristics in the spectral domain.

№	Species	Part	Reflectance (%)		Mean Reflectance	Standard Deviation	Coefficient of Variation	Median	Rate of Change	Delta Reflectance
			Min	Max	*μ*	*σ*	CV	Me	R	∆R
1	Loose smut	General	7.00	13.00	10.00	0.76	7.62	10.00	3.85	6.00
		Healthy	15.00	30.00	22.50	1.91	8.47	22.50	9.62	15.00
		Dried areas	22.50	45.00	33.80	2.86	8.47	33.75	14.42	22.50
		Affected areas	7.00	7.00	7.00	0.00	0.00	7.00	0.00	0.00
2	Head blight	General	22.00	25.00	23.50	0.38	1.62	23.50	1.92	3.00
		Healthy	30.00	45.00	37.50	1.91	5.08	37.50	9.62	15.00
		Dehydrated areas	15.00	22.50	18.80	0.95	5.08	18.75	4.81	7.50
		Affected areas	7.50	7.50	7.50	0.00	0.00	7.50	0.00	0.00
3	FHB	General	32.00	34.00	33.00	0.25	0.77	33.00	1.28	2.00
		Healthy	25.00	40.00	32.50	1.91	5.86	32.50	10.58	15.00
		Dried areas	35.00	48.00	41.50	1.65	3.98	41.50	9.17	13.00
		Affected areas	40.00	60.00	50.00	2.54	5.08	50.00	14.10	20.00
4	Stem rust	General	25.00	32.00	28.50	0.89	3.12	28.50	4.49	7.00
		Healthy	33.00	40.00	36.50	0.89	2.44	36.50	4.49	7.00
		Dried areas	30.00	58.00	44.00	3.56	8.08	44.00	17.95	28.00
		Affected areas	15.00	20.00	17.50	0.64	3.63	17.50	3.21	5.00
5	Net blotch	General	10.00	23.00	16.50	1.65	10.01	16.50	8.33	13.00
		Dried areas	22.00	45.00	33.50	2.92	8.72	33.50	14.74	23.00
		Dehydrated areas	10.00	20.00	15.00	1.27	8.47	15.00	6.41	10.00
		Affected areas	7.50	8.00	7.80	0.06	0.82	7.75	0.32	0.50
6	Spot blotch	General	10.00	24.00	17.00	1.78	10.46	17.00	8.97	14.00
		Dried areas	25.00	50.00	37.50	3.18	8.47	37.50	16.03	25.00
		Dehydrated areas	10.00	25.00	17.50	1.91	10.89	17.50	10.58	15.00
		Affected areas	5.00	10.00	7.50	0.64	8.47	7.50	3.21	5.00
7	Common root rot	General	17.00	31.00	24.00	1.78	7.41	24.00	9.33	14.00
		Healthy	20.00	65.00	42.50	5.72	13.45	42.50	28.85	45.00
		Affected areas	8.00	10.00	9.00	0.25	2.82	9.00	1.28	2.00

**Table 5:** Wavelength parameters of barley spectra.

№	Species	Part	Wavelength (nm)		Spectral Bandwidth	Contrast Ratio	Relative Bandwidth	Normalised Contrast
			Min	Max	SB	C	B_rel_	I_norm_
1	Loose smut	General, Healthy, Dried areas	500	780	280	1.56	0.219	0.359
		Affected areas	500	750	250	1.50	0.200	0.333
2	Head blight	General, healthy, dehydrated and affected areas	500	780	280	1.56	0.219	0.359
3	FHB	General	500	780	280	1.56	0.219	0.359
		Healthy, dried and affected areas	550	780	230	1.42	0.173	0.295
4	Stem rust	General, healthy, dried and affected areas	500	780	280	1.56	0.219	0.359
5	Net blotch	General, healthy, dried and affected areas	500	780	280	1.56	0.219	0.359
6	Spot blotch	General, dried and affected areas	500	780	280	1.56	0.219	0.359
		Dehydrated areas	550	780	230	1.42	0.173	0.295
7	Common root rot	General	400	600	200	1.50	0.200	0.333
		Healthy, affected areas	500	780	280	1.56	0.219	0.359

**Table 6:** Model performance statistics.

Algorithm	Macro Accuracy (R^2^Y)	Cross Validation Macro Accuracy (Q^2^Y)	Log Loss	Log Loss Reduction	Micro Accuracy	Macro Accuracy Test
Neural Network (AP)	0.70465	0.66924	0.50097	0.69462	0.78914	0.74545
SVM	0.68392	0.61869	0.56442	0.65594	0.76200	0.68986
Random Forest (Fast Forest)	0.81112	0.78784	0.42022	0.73772	0.85178	0.84644
Maximum Entropy (SDCA)	0.74279	0.72188	0.45819	0.71424	0.81058	0.73506
SIMCA	0.84660	0.83969	-	-	-	-

**Table 7:** Metrics of performance and quality for machine learning algorithms.

Metrics	Loose Smut	Head Blight	FHB	Stem Rust	Net Blotch	Spot Blotch	Common Root Rot
Neural Network (AP)							
Precision	100.00	0.00	97.60	95.90	91.70	91.50	100.00
Recall	51.90	0.00	95.40	87.50	11.30	30.60	55.20
F1-score	68.40	0.00	96.50	91.50	20.10	45.80	71.10
Binary Accuracy	51.95	0.00	95.40	87.50	11.31	30.56	55.17
Balanced Accuracy	75.95	50.00	97.36	93.33	55.51	64.76	77.60
MCC	0.704	-	0.955	0.899	0.283	0.455	0.742
SVM							
Precision	100.00	0.00	98.00	98.10	0.00	94.20	100.00
Recall	3.25	0.00	93.30	84.00	0.00	12.60	20.70
F1-score	6.29	0.00	95.60	90.50	0.00	22.20	34.30
Binary Accuracy	3.25	0.00	93.32	83.97	0.00	12.56	20.69
Balanced Accuracy	51.63	50.00	96.37	91.82	50.00	56.15	60.35
MCC	0.172	-	0.944	0.890	-	0.291	0.454
Random Forest (Fast Forest)							
Precision	99.30	100.00	99.40	99.00	91.10	91.20	100.00
Recall	98.90	35.60	96.20	95.60	65.20	75.20	100.00
F1-score	99.10	52.50	97.80	97.30	76.00	82.40	100.00
Binary Accuracy	98.92	35.56	96.18	95.62	65.25	75.16	100.00
Balanced Accuracy	99.42	67.80	98.01	97.69	81.75	86.21	100.00
MCC	0.990	0.595	0.971	0.967	0.724	0.772	1.000
Maximum Entropy (SDCA)							
Precision	100.00	0.00	98.90	98.90	85.70	87.40	100.00
Recall	97.60	0.00	95.70	94.10	38.90	57.20	93.10
F1-score	98.80	0.00	97.30	96.40	53.50	69.10	96.40
Binary Accuracy	97.62	0.00	95.66	94.12	38.92	57.16	93.10
Balanced Accuracy	98.80	50.00	97.70	96.93	68.58	77.02	96.55
MCC	0.987	-	0.965	0.957	0.516	0.627	0.965
SIMCA							
Precision	10.60	0.00	100.00	100.00	56.90	74.50	100.00
Recall	99.80	0.00	17.00	2.78	18.40	8.68	96.60
F1-score	19.10	0.00	29.10	5.41	27.80	15.50	98.20
Binary Accuracy	99.78	0.00	17.00	2.78	18.35	8.68	96.55
Balanced Accuracy	58.08	50.00	58.50	51.39	57.34	53.77	98.30
MCC	0.130	-	0.370	0.151	0.237	0.191	0.983

**Table 8:** Formulas and interpretation of object spectral characteristics [63,74,75,76,77,78,79,80].

No.	Indicator	Formula	Interpretation
1	Explained Variance	${Explained\;Variance}_{i}\left(\%\right)=$ $=\frac{\lambda_{i}}{\sum_{j=1}^{m}\lambda_{j}}\times 100\%$	Contribution of the *i*-th component to total data variability
2	Cumulative Variance	${Cumulative\;Variance}_{k}\left(\%\right)=$ $=\sum_{i=1}^{k}\frac{\lambda_{i}}{\sum_{j=1}^{m}\lambda_{j}}\times 100\%$	Cumulative proportion of variance accumulated up to the *k*-th component
3	Maximum reflectance	$R_{max}=max(R_{1},R_{2},\ldots ,R_{n})$	Range of reflectance coefficient values (from minimum to maximum)
4	Minimum reflectance	$R_{min}=min(R_{1},R_{2},\ldots ,R_{n})$	
5	Mean reflectance coefficient	$\mu =\frac{1}{n}\sum_{i=1}^{n}R_{i}$,	Mean value of the reflected signal
6	Standard deviation	$\sigma =\sqrt{\frac{1}{n}\sum_{i=1}^{n}{{(R}_{i}-\mu )}^{2}}$	Degree of reflectance deviation from the mean level
7	Coefficient of variation	$CV=\frac{\sigma }{\mu }\times 100$	Level of spectral data variability
8	Rate of reflectance change	$R=\frac{R_{2}-R_{1}}{\lambda_{2}-\lambda_{1}}$	Nature of variations within the spectrum
9	Delta reflectance	$∆R=R_{max}-R_{min}$	Amplitude of reflectance change across the considered range
10	Spectral bandwidth	$SB=\lambda_{max}-\lambda_{min}$	Coverage of the investigated spectral region
11	Contrast ratio	$C=\frac{x_{max}}{x_{min}}\;(x_{min}\neq 0)$	Ratio of maximum response to minimum value
12	Relative bandwidth	$B_{rel}=\frac{x_{max}-x_{min}}{x_{max}+x_{min}}$	Indicator of spectral heterogeneity independent of absolute intensity
13	Normalised contrast	$I_{norm}=\frac{x_{max}-x_{min}}{x_{max}}$	Measure of signal variability irrespective of its overall level
14	Precision	$Precision=\frac{TP}{TP+FP}$	Proportion of correctly identified positive results among all positive predictions
15	Recall	$Recall=\frac{TP}{TP+FN}$	Model’s ability to detect objects of the target class
16	F1-score	$F1=2*\frac{Precision*Recall}{Precision+Recall}$	Balanced metric combining precision and recall
17	Binary Accuracy	$Accuracy=\frac{TP+TN}{TP+TN+FP+FNl}$	Proportion of all correctly classified instances among total observations
18	Balanced Accuracy	$BA=\frac{1}{2}*\left(\frac{TP}{TP+FN}+\frac{TN}{TN+FP}\right)$	Average ability of the model to correctly classify each class regardless of class size
19	MCC	MCC= $\frac{TP*TN-FP*FN}{\sqrt{\left(TP+FP\right)\left(TP+FN\right)(TN+FP)(TN+FN)}}$	Classification quality assessment metric; correlation between true and predicted labels

## Data Availability

The data supporting this study’s findings are available on request from the corresponding author.
